# Recent advances in natural polysaccharides for type 2 diabetes management: sources, structural characteristics, and mechanistic insights

**DOI:** 10.3389/fphar.2025.1705122

**Published:** 2025-12-19

**Authors:** Zexiang Liu, Hanlong Wang, Xiujuan Gong, Xiao Jiang, Kangyi Yang, Linfang Jiang, Zhihui Wang, Qiaozhen Tong

**Affiliations:** 1 School of Pharmacy, Hunan University of Chinese Medicine, Changsha, China; 2 Hunan Provincial Key Laboratory of Germplasm Resources and Standardized Cultivation of Bulk GentineMedicinal Materials, Changsha, China

**Keywords:** therapeutic polysaccharides of natural origin, type 2 diabetes mellitus, structural characterization, complications of type 2 diabetes mellitus, mechanism

## Abstract

The global prevalence of type 2 diabetes mellitus (T2DM) continues to rise, posing significant challenges for existing therapeutic strategies, which are often limited by poor bioavailability, high cost, and adverse effects. Natural polysaccharides have emerged as promising metabolites due to their broad sources, favorable safety profiles, and stable efficacy. This review systematically summarizes the sources, structural characteristics, and mechanisms of action of natural polysaccharides with anti-T2DM activity, with a particular focus on their multi-target regulatory effects in mitigating T2DM and its complications. By integrating structural diversity—such as molecular weight, glycosidic linkage patterns, and branching architecture—with functional outcomes across key pathogenic pathways including insulin resistance, inflammation, oxidative stress, gut microbiota dysbiosis, and mitochondrial dysfunction, this work elucidates fundamental structure-activity relationships. The review not only bridges existing knowledge gaps in multi-mechanistic integration but also provides a conceptual framework for the structure-based development of polysaccharide-based therapeutics against T2DM, highlighting future research directions.

## 1 Introduction

Diabetes mellitus (DM) is a chronic metabolic disorder that significantly impairs patients’ quality of life, poses serious health risks, and has placed a huge economic burden on healthcare systems around the world (Ma et al., 2019). According to the International Diabetes Federation (IDF), the global prevalence of diabetes is projected to reach 642 million cases by 2040 (Weidner et al., 2018), with Type 2 diabetes mellitus (T2DM) constitutes over 90% of all diagnosed cases of diabetes (Wang L. et al., 2021). T2DM, also referred to as non-insulin-dependent DM, is a multifactorial endocrine-metabolic disorder characterized by hyperglycemia and dyslipidemia. This complex condition arises from the interaction between genetic and environmental factors, leading to manifestations such as insulin resistance (IR), pancreatic β-cell dysfunction, gut microbiota dysbiosis, chronic low-grade inflammation and other endocrine disturbances (Hui et al., 2019). The main pathophysiological feature of T2DM is insulin resistance accompanied by a relative deficiency in insulin secretion, which ultimately results in disrupted glucose homeostasis (Wu et al., 2016). Inadequate management of T2DM can lead to severe complications, including diabetic microvascular complications, diabetes-associated coronary heart disease, diabetic nephropathy, diabetic fatty liver disease, and diabetic retinopathy, all of which significantly threaten human health and life expectancy (Spohn et al., 2014). Current therapeutic strategies for diabetes management primarily involve oral hypoglycemic agents and insulin therapy. The main types of antidiabetic drugs include insulin, thiazolidinediones, metformin/guanidine derivatives, sulfonylureas, α-glucosidase inhibitors, meglitinides, sodium-glucose cotransporter-2 (SGLT2) inhibitors, glucagon-like peptide-1 (GLP-1) receptor agonists, and dipeptidyl peptidase-4 (DPP-4) inhibitors (Brendan et al., 2018). However, long-term use these pharmacological agents may result in adverse effects, drug tolerance, and may not effectively prevent disease progression and complications (Hui et al., 2019). Consequently, there is an immediate need for developing effective, harmless, and cost-efficient therapeutic agents for DM management. The most recent studies have highlighted the considerable therapeutic potential of polysaccharides in the treatment of T2DM (Wang et al., 2016). As natural macromolecules, polysaccharides have garnered considerable attention in life sciences and pharmaceutical research due to their diverse biological activities, including immunomodulatory, antitumor, antioxidant, and hypoglycemic effects. Accumulating research suggests that therapeutic polysaccharides of natural origin, serving as key bioactive metabolites in botanical drugs, exhibit remarkable antidiabetic properties with minimal side effects or adverse drug reactions (Wu et al., 2016). Polysaccharides, which are complex carbohydrate polymers with the general formula C_x_ (H_2_O)_y_ are complex carbohydrate polymers formed by the connection of monosaccharide units through glycosidic bonds. Upon hydrolysis, these polymers yield monosaccharides or oligosaccharides, primarily including glucose, galactose, mannose, arabinose, fructose, rhamnose, and xylose. Given that the repeating units in polysaccharide backbones are typically hexose monosaccharides, their general formula is often expressed as (C_6_H_10_O_5_)_n_, where n ranges between 40 and 3,000 (Cao et al., 2012). The structural complexity and compositional diversity of herbal polysaccharides confer several therapeutic advantages, including multi-target activity, reduced adverse reactions, and wide availability for disease treatment (Zho et al., 2023).

Numerous reviews have explored the potential of natural polysaccharides in managing metabolic diseases, however, most prior studies have primarily focused on general biological activities or isolated mechanisms, such as antioxidant or immunomodulatory effects (Shen et al., 2024). For instance, while existing research summarizes the hypoglycemic properties of plant-derived polysaccharides or their role in regulating the gut microbiota (Zhao et al., 2024), a systematic integration of multi-mechanistic pathways is often lacking. This is particularly evident regarding the connections between detailed structural characterization, structure-activity relationships, and their impact on T2DM complications.

In contrast, this review provides a comprehensive and systematic analysis of polysaccharides derived from plants, algae, and fungi, emphasizing the critical link between their structural diversity and corresponding mechanisms of action in alleviating T2DM and its complications. By integrating recent advances in structural analysis techniques, this study links these polysaccharides to functional outcomes across multiple pathogenic pathways—including insulin resistance, inflammation, oxidative stress, dysbiosis, and mitochondrial dysfunction. This unique, mechanism-centric, and holistic approach aims to bridge existing knowledge gaps and establish a foundational framework for the future development of structure-optimized polysaccharide therapeutics against T2DM.

## 2 Sources of therapeutic polysaccharides of natural origin

**FIGURE 1:**
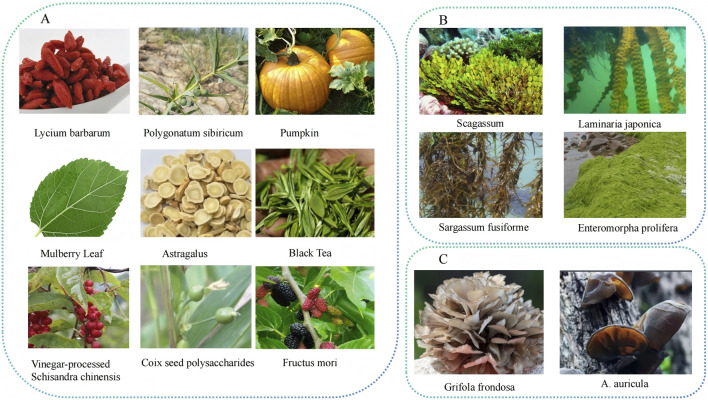
Schematic overview of natural sources of polysaccharides with anti-T2DM activity. This diagram categorizes common natural sources yielding polysaccharides investigated for their potential in managing Type 2 Diabetes Mellitus (T2DM). **(A)** Vegetal Species: Represents a selection of medicinal and edible plants known for their bioactive polysaccharides, including Lycium barbarum (Goji berry), Polygonatum sibiricum, Pumpkin, Mulberry Leaf, Astragalus membranaceus, Black Tea, Vinegar-processed Schisandra chinensis, Coix seed, and Fructus mori (Mulberry fruit). **(B)** Marine Algae: Highlights macroalgae species such as Sargassum, Sargassum fusiforme, and Enteromorpha prolifera, which are rich in unique sulfated polysaccharides. **(C)** Fungi: Features medicinal mushrooms like Grifola frondosa (Maitake) and Auricularia auricula (Wood ear), whose fruiting bodies contain immunomodulatory and metabolic-regulating polysaccharides. This figure underscores the taxonomic diversity of natural organisms serving as promising reservoirs for the development of polysaccharide-based T2DM therapeutics or functional foods.

Polysaccharides are a class of macromolecules with rich structure, abundant sources, and multiple physiological activities, which have broad application prospects in functional foods as well as in the area of medicine and health (Xie et al., 2016). Recent studies have shown that polysaccharides derived from various natural medicines exhibit distinct structural specifications and physicochemical properties. The relationship between the structure and function of polysaccharides in food processing is crucial for the advancement of new foods and pharmaceuticals. This section reviews natural polysaccharides from various sources relevant to T2DM management (Figure 1). It aims to elucidate the mechanism of their action in alleviating T2DM and provide new ideas for the advancement of anti-T2DM drug research.

### 2.1 Vegetal species

Botanical drugs derived from vegetal species are highly valued for their unique physiological activities (Wan et al., 2024). Among these polysaccharide metabolites, plant-derived polysaccharides show great potential for application in dietary intervention and disease treatment, and they are considered as highly promising metabolites for replacing conventional therapeutic drugs (Kuang et al., 2023). In recent years, more and more studies have shown that traditional Chinese botanical drugs, recognized as a potential hypoglycemic agent, is gradually gaining acceptance within the medical community. Furthermore, a growing array of natural medicinal vegetal species is being investigated and utilized in contemporary pharmacological research to elucidate their therapeutic value (Wang H. et al., 2021). Multiple studies have shown that natural plant polysaccharides are able to have anti-diabetic effects by affecting various mechanisms such as IR, intestinal flora, and inflammation (Bo et al., 2024).

*Lycium chinense* Mill*.* are a medicinal and edible plant rich in various bioactive polysaccharide metabolites, which play a significant role in alleviating T2DM. A research study has found that Lycium barbarum polysaccharide (LBP) has a significant protective effect on T2DM patients (Cai et al., 2015). After 3 months of LBP supplementation, blood glucose levels decreased significantly, insulin production indices increased, and LBP also elevated high-density lipoprotein (HDL) levels in T2DM patients. Compared to patients taking hypoglycemic medications, LBP demonstrated more pronounced hypoglycemic effects in patients not taking any hypoglycemic medications. Research shows that *Polygonatum sibiricum* Redouté polysaccharide (PSP) reduces IR indices in T2DM mice, increases oral glucose tolerance test (OGTT) and serum insulin levels, reduce free fatty acid content to enhancement lipid metabolism, and reduces glycated serum protein levels to promote glucose metabolism, therefore lowering blood glucose concentrations (Bo et al., 2024). Additionally, PSP has a reparative effect on impaired liver tissue cells and pancreatic tissue in T2DM mice (Wang et al., 2024). Researchers have discovered that *Cucurbita moschata* polysaccharide (CMP) can improve insulin tolerance, lower blood glucose (GLU), total cholesterol (TC), and low-density lipoprotein (LDL-C) levels, meanwhile increasing HDL levels, thereby alleviating T2DM (Cai et al., 2015). Furthermore, CMP modulates the gut microbiota structure and selectively enriches key bacterial genera such as *Bacteroides*, *Precursor bacterium genus*, *Deltaproteobacteria*, *Oscillospira*, *Veillonellaceae*, *Phascolarctobacterium*, *Sutterella*, and *Bilophila*, thereby alleviating T2DM (Liu et al., 2018). Studies have demonstrated that *Morus alba* L. Leaf Polysaccharide (MLP) significantly improves oral glucose tolerance in diabetic rats, restores glycogen levels, and enhances glucose synthase (GS) activity (Ren et al., 2015). Insulin resistance in diabetic rats treated with MLP was also improved. Additionally, the expression levels of insulin receptor substrate 2 (IRS2), phosphoinositide 3-kinase (PI3K), and protein kinase B (PKB/AKT), which are involved in insulin signal transduction, were significantly increased, while the expression level of protein tyrosine phosphatase 1B (PTP1B) was significantly decreased. In the MLP-treated group, the levels of 8-hydroxy-2-deoxyguanosine (8-OHdG) and malondialdehyde (MDA) in the liver were significantly reduced, while the activity of antioxidant enzymes such as superoxide dismutase (SOD), glutathione peroxidase (GPx), and catalase (CAT) was significantly increased. These results clearly indicate that MLP inhibits PTP1B expression, activates the phosphatidylinositol 3-kinase (PI3K-AKT) pathway, alleviates oxidative stress in the livers of high-fat and streptozotocin (STZ)-induced T2DM rats, and regulates liver glucose metabolism and insulin signaling effectively (Ren et al., 2015). Other research shows that the primary effects of *Astragalus* Polysaccharides (APS) on diabetes are to reduce IR, promote pancreatic cell proliferation, inhibit pancreatic β-cell death, and thereby improve diabetic symptoms (Xu et al., 2025). *Camellia sinensis* (L.) Kuntze Polysaccharides (CSP) have potential anti-diabetic effects. Zhang Z. et al. (2024) study showed that oral administration of CSP effectively reduces fasting blood glucose (FBG) levels in T2DM mice, upregulates p-Akt/p-PI3K expression, and significantly promotes the translocation of glucose transporter 2 (GLUT2) in the liver, thereby alleviating hyperglycemia. Research shows that vinegar-processed *Schisandra chinensis* (Turcz.) Baill. polysaccharide (VSP) has a significant therapeutic effect on T2DM mice, regulating imbalanced glucose and lipid metabolism, alleviating pancreatic and hepatic damage, restoring intestinal barrier integrity, and inhibiting inflammatory responses (Guo et al., 2025). Serum metabolomics and microbiological analyses revealed that VSP significantly regulates 104 endogenous metabolites, correcting intestinal microbiota dysbiosis in T2DM mice. Additionally, VSP increases short-chain fatty acid (SCFA) levels and GPR41/43 expression in the colon of T2DM mice, thereby alleviating related symptoms in T2DM patients, providing a foundation for further development of VSP (Xu et al., 2025). Xia et al. (2021) found that *Coix lacryma-jobi* L. polysaccharides (CLP) can increase serum insulin and HDL cholesterol levels in T2DM mice, reduce TC, triglyceride (TG), and LDL-C levels, while CLP treatment helps repair the intestinal barrier and regulate the intestinal microbiota composition in T2DM mice. It also promotes the growth of SCFA-producing bacteria, activates the IGF1/PI3K/AKT signaling pathway, and thereby lowers blood glucose levels in T2DM mice (Xia et al., 2021). Some scholars have discovered that Fructus *Morus alba* L. polysaccharide (FMP) can alleviate hyperglycemia, endotoxemia, hyperlipidemia, IR, and high metabolic inflammation levels in T2DM mice (Chen X. et al., 2023). FMP can also significantly inhibit endotoxin-producing *Shigella* bacteria, promote the widespread application of probiotics *Lactobacillus* and *Bifidobacterium*, and thereby repair the intestinal barrier to alleviate symptoms in T2DM mice (Chen X. et al., 2023).

To sum up, comparing polysaccharide components from different sources reveals their shared capacity to counteract diabetes. However, their mechanisms of action primarily involve ameliorating diabetic symptoms through enhancing insulin sensitivity, retarding carbohydrate digestion and absorption, regulating the activity of enzymes involved in glucose metabolism, and protecting pancreatic β-cell function. Regarding efficacy, the potency and mechanisms of action against diabetes may vary among different polysaccharides due to differences in composition and purity. Interestingly, the plants yielding these polysaccharides possess dual medicinal and edible properties, positioning their polysaccharides as promising metabolites for functional foods and pharmaceutical development. Incorporating polysaccharide-based adjunctive therapies into conventional T2DM management can significantly enhance pharmacological efficacy, offering novel therapeutic benefits for patients. Moreover, the integration of plant-based ingredients within traditional dietary practices resonates with modern food science’s precise investigation of bioactive metabolites. This convergence provides multidimensional evidence supporting the potential of plant polysaccharides to alleviate T2DM and mitigate associated complications.

### 2.2 Marine algae

Marine species are rich in various bioactive metabolites and possess significant potential for pharmaceutical development and application (Thao et al., 2015). Algae are a group of lower autotrophic organisms widely distributed in marine and freshwater ecosystems, characterized by extremely high species diversity and ecological adaptability. To date, over 1,000 species of algae have been identified. Ingestible aquatic macroalgae can be classified into three distinct categories: *Laminaria japonica, Undaria pinnatifida, and Sargassum fusiforme* (Chen D. et al., 2022). Seaweed is a rich source of polysaccharides, which serve as the primary components of cell wall structure and energy reserves, accounting for over 50% of the dry weight of seaweed (Pluvinage et al., 2018). Since most marine macroalgae thrive in harsh natural conditions characterized by high pressure, high salinity, low temperatures, and limited light exposure, they adapt to these extreme marine environments by producing unique secondary metabolites, including polysaccharides (Wang Y. et al., 2022). These macroalgal polysaccharides exhibit diverse biological activity and medicinal functions, including anti-diabetic (Fang F. et al., 2022), anti-inflammatory, anti-coagulant, anti-oxidant, anti-viral, anti-tumor, anti-apoptotic, anti-proliferative, and immune-stimulating properties (Teng et al., 2025).

Recent studies show that used a hot water extraction method to extract three types of *algae* polysaccharides (APs) from *Macrocystis pyrifera*, *Sargassum fusiforme*, and *Sargassum* spp., which significantly inhibited weight loss in diabetic rats and increased their water intake, while effectively controlling elevated blood glucose, TG, TC levels in diabetic rats (Jia et al., 2020). There are even studies showing that the *Gracilaria lemaneiformis* polysaccharide extracted from *Gracilaria lemaneiformis* (Bory) P.C.Silva (GLP), GLP has hypoglycemic and antioxidant effects on diabetic mice, significantly enhancing the activity of antioxidant enzymes (SOD and GSH-Px) and total antioxidant capacity (T-AOC) in the liver, pancreas, and kidneys of diabetic mice, and exhibiting a certain degree of repairing the kidneys and pancreas (Liao et al., 2015). Tong et al. (2024) found that *Laminaria japonica* polysaccharide (LJO) significantly reduced FBG levels, insulin levels, and inflammatory factors in T2DM mice. Furthermore, LJO upregulated the projection of insulin receptor substrate 1 (IRS-1), thereby alleviating IR and mitigating T2DM levels. Researchers (Lin et al., 2024) found that a water-soluble polysaccharide (EP) purified from the edible algae *Ulva prolifera* O.F. Müller effectively reduced blood glucose, liver index, epididymal fat index, serum TC, and TG in diabetic mice, while promoting liver glycogen synthesis and alleviating diabetic symptoms.

Among the planet’s naturally occurring reserves, marine algae-which include both the larger macroalgae and microscopic microalgae–constitute one of the richest and most uniquely specialized assets (Li et al., 2022). Although marine algae-derived polysaccharides demonstrate compelling application potential in combating T2DM due to their multi-targeted, multifunctional mechanisms of action, their clinical translation remains constrained by knowledge gaps in critical therapeutic pathways. On the one hand, existing research has confirmed that these polysaccharides exert effects through pathways such as regulating insulin sensitivity, inhibiting key enzymes in glucose metabolism, and improving gut microbiota imbalance; yet the core question of how structure determines function remains unresolved. For instance, no clear patterns have emerged linking structural characteristics-such as molecular weight, monosaccharide composition, or glycosidic bond types-to anti-T2DM activity, directly hindering their transition from laboratory research to practical application. To overcome this bottleneck, it is imperative to establish a research framework utilizing advanced glycomics analytical techniques. Glycomics tools such as high-resolution mass spectrometry and high-performance liquid chromatography can precisely elucidate the intricate chemical structures of algal polysaccharides, identifying the core composition of their active moieties. Concurrently, computational biology techniques such as molecular docking and molecular dynamics simulations should be integrated to model, at the molecular level, the interaction patterns between polysaccharides and target proteins (e.g., insulin receptors, α-glucosidase), thereby elucidating the intrinsic mechanisms underlying the ‘structure-activity relationship.’ Therefore, gaining a deeper understanding of the precise pathways through which algal polysaccharides exert therapeutic effects on T2DM necessitates more precise and advanced research.

### 2.3 Fungi

Fungal polysaccharides are important bioactive metabolites extracted from fungal fermentation broth, mycelium, and fruiting bodies, and they are a key focus of research. They are long-chain carbohydrates composed of all kinds of neutral sugars or aldehydes linked by glycosidic bonds (Liang et al., 2021), demonstrating a variety of biological functions, including immune modulation, antioxidant activity, and anti-tumor effects, and they have widespread applications in medicine, life sciences, and the food industry (Dong et al., 2022). Edible fungi belong to the category of potential plant-based therapies for diabetes. Abundant in naturally occurring constituents like fiber, polysaccharides, phenols, and alkaloids, these organisms have long been valued for their efficacy in combating diabetes, countering oxidative stress, and managing elevated lipid levels. Contemporary investigations further demonstrate that polysaccharides derived from fungi offer distinct therapeutic benefits for managing T2DM (Khursheed et al., 2020).

Research has found that treatment with *Grifola frondosa* (Dicks.) Gray polysaccharide (GFP) reduced FBG levels in diabetic mice, enhanced oral glucose tolerance, eased IR, protected the liver and kidneys from damage, and reduced inflammation (Chen et al., 2019). Additionally, it improved hepatic IR by regulating the IRS1/PI3K and JNK signaling pathways, thereby playing a role in alleviating T2DM (Chen et al., 2019). Recent research indicated that *Auricularia auricula* (L.ex Hook.) Underw polysaccharides (AAPs) significantly reduced inflammation, liver damage, and IR (Xu et al., 2021). Furthermore, AAPs improve glucose and lipid metabolism disorders by activating the AKT and AMPK signaling pathways in T2DM mice, thereby adjusting the gut microbiota (Xu et al., 2021). Udomlak et al. (2025) found that polysaccharides extracted from *Schizophyllum commune* can increase insulin and GLUT2 levels in T2DM mice and reduce MDA expression, thereby alleviating T2DM to some extent. And there are also studies that have found that *Inonotus obliquus* Pilát polysaccharides (IOPs) can lower TG levels, dramatically recover body weight and fat mass, decline FBG levels, enhance glucose tolerance, rise liver glycogen levels, improve IR, and alleviate STZ-induced damage to organ tissues (liver, kidneys, and pancreas) (Wang et al., 2017). Additionally, after IOPs treatment, the protein expression of PI3K-p85, p-Akt (ser473), and glucose transporter protein 4 (GLUT4) was upregulated, suggesting that the anti-hyperglycemic mechanism of IOPs may involve the activation of PI3K and Akt phosphorylation, and GLUT4 transport in diabetic mice (Wang et al., 2017). In addition, research has found that *Pleurotus ostreatus* (Jacq.) P. Kumm. polysaccharides (POP) extracted from oyster mushrooms can reduce hyperglycemia and hyperlipidemia in T2DM rats, improve IR, and increase glycogen storage (Zhang et al., 2016). Additionally, POP can increase the activity of GSH-Px, CAT, and SOD while reducing MDA levels to mitigate oxidative damage risk, thereby alleviating T2DM-related symptoms (Zhang et al., 2016).

This fully demonstrates the immense potential of fungal polysaccharides in the field of T2DM intervention. Compared to polysaccharides from other sources, fungal polysaccharides possess distinct advantages. Firstly, they predominantly originate from medicinal fungi with dual food and medicinal properties, whose long-term safety has been validated by both traditional practice and modern toxicological research, resulting in higher patient acceptance. Secondly, fungal polysaccharides exhibit greater structural complexity and diversity. Beyond common glucans, they encompass specialized structures such as galactans and mannans. This structural variety enables them to regulate glucose metabolism through a broader array of pathways, offering increased possibilities for T2DM intervention. However, unlike certain water-soluble seaweed polysaccharides, most fungal polysaccharides suffer from poor water solubility, susceptibility to degradation *in vivo*, and low bioavailability. Conventional extraction methods struggle to isolate highly active specific components, leading to significant variability in polysaccharide activity across studies and compromising result reproducibility. Concurrently, existing clinical studies predominantly focus on “single polysaccharides” as intervention targets, neglecting the fact that fungal polysaccharides often exert effects in “formulae” within traditional applications. The clinical efficacy disparity between “single components” and “formulae synergism” remains unresolved, hindering the establishment of standardized clinical protocols. Consequently, subsequent research on fungal polysaccharides must simultaneously: -Deepen understanding of their unique mechanisms distinct from other polysaccharides; -Optimize extraction techniques and design novel formulations; -Conduct compound studies and individualized trials more closely aligned with clinical practice. Only through this integrated approach can fungal polysaccharides transition from fundamental research to clinical intervention in T2DM management.

## 3 Structural characterization

The molecular structure of polysaccharides serves as the material basis for their hypoglycemic activity. Accurately defining the molecular architecture of polysaccharides, including fundamental parameters like molecular weight and monosaccharide profile, along with complex features such as glycosylation motifs, branching patterns (encompassing chain length and position), substituent characteristics, and three-dimensional spatial arrangements plays a pivotal role in deciphering how structural variations govern these compounds’ therapeutic impact on T2DM (Chen S.-K. et al., 2023). Recent advancements have driven the adoption of diverse analytical techniques for polysaccharide structural elucidation, spanning from conventional methods like Fourier transform infrared spectroscopy (FT-IR) to sophisticated chromatography platforms-including gel permeation (GPC), high-performance liquid (HPLC), gas (GC), high-pressure size-exclusion (HPSEC), and high-pressure anion exchange chromatography (HPAEC)-alongside hyphenated systems such as gas chromatography-mass spectrometry (GC-MS) and core spectroscopic tools like nuclear magnetic resonance (NMR). The structural characteristics of natural polysaccharides with anti-T2DM activity are summarized in Table 1.

**TABLE 1:** Structural characterization of natural polysaccharides with anti-T2DM effects.

Source	Name	Compound name	Carbohydrate composition	Molecular mass (KDa)	Structure	Mechanism	References
Vegetal species	Polysaccharide from *Momordica charantia* L. A	MCPS-3	Rhamnose, glucuronic acid, galacturonic acid, glucose, galactose, and arabinose in molar ratios of 10.66:3.66:258.0:1.0:51.0:9.338	93.796	------	Activates IRS1/PI3K/Akt and AMPK signaling pathways to improve insulin sensitivity, enhance glycolysis, and increase glycogen synthesis. Increases beneficial flora and enhances microbial diversity to regulate gut flora composition	Zhang et al. (2025)
	Polysaccharide from *Abelmoschus esculentus* L. Moench	AeP-P-1	L-rhamnose, D-galactose and D-galacturonic acid with the ratio of 1.87:3.58:1.00	3.02	T-linked-Rhap, T-linked-Galp, 1,2,4-linked-Rhap, 1,4-linkedGalp, 1,6-linked-Galp, and 1,3,4-linked-Galp	Regulation of the insulin/PI3K/AKT pathway lowers blood glucose by enhancing GSK3β phosphorylation and decreasing GSK3β activity, maintaining GCS and gluconeogenesis	Geng et al. (2022)
	*Cucurbita moschata* Duchesne polysaccharide	PWESP-3	Arabinose (Ara, 35.30 mol%), galactose (Gal, 61.20 mol%), glucose (Glc, 1.80 mol%), and Mannuronic acid (ManA, 1.70 mol%)	140.519	Araf-(1→, →3)-Araf-(1→, →5)-Araf-(1→, Glcp-(1→, Galp-(1→, →3,5)-Araf-(1→, →2)-Glcp-(1→, →2)-Manp-(1→, →3)-Glcp-(1→, →4)-Galp-(1→, →3)-Galp-(1→, →6)-Galp-(1→, →3,4)-Galp-(1→, →4,6)-Galp-(1→, →residues in the backbone)	Improves insulin secretion and reduces oxidative stress levels	Zhang et al. (2024b)
	*Astragalus membranaceus* (Fisch.) Bung-e polysaccha-ride-D1	APS-D1	Galactose and arabinose with a molar ratio of 104.38:1.67:1 (97.51%:1.56%:0.93%)	7.36	→4)-α-D-Glcp-(1→ residue backbone with →3)-β-D-Galp-(1→ residue and terminal-α/β-D-Glcp-(1→ side chains	Improves insulin dysfunction and IR, and ameliorates immune and inflammatory conditions as well as pancreatic and liver tissue damage	Long et al. (2024)
	*Cornus officin-alis*	PFC-CI	Rha, GalA, Gal, and Ara in a molar ratio of 2.04:14.01:1.00:6.38	59.0	T-α-Galp-(1→6)-α-Galp-(1→6)-α-Galp-(1→[4)-GalpA-(1→4)-GalpA-OMe-(1→4)- α-GalpA-3-OAc-(1→]m→[2)-Rhap-(1→4)-α-GalpA-(1→]n, with side chains composed of α-Araf-(1→, →3)-α-Araf-(1→, →3,5)-α-Araf-(1→, →5)-α-Araf-(1→	Modulation of the glucose transporter protein 2 (Glut 2)/glucokinase signaling pathway exerts anti-T2DM effects and attenuates IR.	Ma et al. (2025)
	*Gynostemma pentaphyllum* (Thunb.) Makino	GPP	Rhamnose, arabinose, galactose, glucose, xylose, mannose, galacturonic acid and glucuronic acid in a molar ratio of 4.11:7.34:13.31:20.99:1.07:0.91:4.75:0.36	40.70	------	Inhibition of glucose uptake and influence on protein expression of GLUT2	Wang et al. (2019a)
	*Bletilla striat-a*	BSP1-1	Man and Glc (molar ratio was 3.68:1.00)	22.65	→4)-β-D-Man-(1→, →4)-α-DMan-(1→, →4)-β-D-Glc-(1→, →4)-β-D-Glc-(1→)	Upregulation of Nrf2 and its downstream antioxidant HO-1 alleviates oxidative stress and promotes wound healing, and ameliorates T2DM-related conditions	Li et al. (2025)
	*apricot (Armeniaca Sibirica L. Lam) ker-nels Prunus sibiric-a*	AP-1	Glucose with trace amounts of arabinose, galactose, and mannose, which had molar percentages of 98.48, 0.63, 0.62% and 0.27%	23.408	AP-1 was composed of →4)-α-D-Glcp-(1 → interlinked, and α-D-Glcp-(1 → was attached as a branched chain at the O-6 position of →4,6)-α-D-Glcp-(1→. In addition, AP-1 exhibited stronger α-glucosidase	Inhibits glucosidase and enhances free radical scavenging	Peng et al. (2023)
	*Hemerocallis citrina*	HCBP-1	Glucose, galactose, xylose, rhamnose, mannose, galacturonic acid and glucuronic acid with a molar ratio of 58:56:43:17:7:5:1	16.68	------	Enhancement of antioxidant enzyme activities and reduction of MDA levels lead to attenuation of oxidative stress damage and improvement of glucose intolerance and IR in T2DM patients	Ti et al. (2022)
		HCBP-2	Galactose, glucose, galacturonic acid, xylose, glucuronic acid, arabinose and mannosewith a molar ratio of 18:10:9:6:3:2:1	1.45	------		
		HCBP-3	Galactose, galacturonic acid, xylose, glucose, glucuronic acid, arabinose and mannose with a molar ratio of 15:12:9:7:6:2:1	42.29	------		
	*Inula japonica* Thunb	IJP-B-1	Glucose, arabinose, galactose, mannose, rhamnose, xylose and galactocuronic acid	37	(1→3, 6)-linked-galactose and other branched residues	Protects β cells and fights oxidative stress	Zhao et al. (2017)
	*Hovenia dulci-s*	HDP-2A	Man, Rha, GlcA, GalA, Glc, Gal, and Ara at a molar ratio of 3.64:1.41:4.67:5.16:3.01:60.02:22.09	372.91	HDPs-2A is composed primarily of →5)-α-L-Araf-(1→, →5)-α-L-Araf-(1→, →3,5)-α-L-Araf-(1→, →6)-β-D-Galp-(1→, →3,6)-β-D-Galp-(1→, T-β-D-Galp, →3)-β-D-Galp-(1→, and T-α-D-Glcp	Improves symptoms of hyperglycemia and dyslipidemia and improves insulin secretion	Yang et al. (2022)
	*Rehmannia gl-utinosa* (Gaertn.) DC.	RGP	Rhamnose, arabinose, mannose, glucose and galactose in the molar ratio of 1.00:1.26:0.73:16.45:30.40	63.5	------	Increased basal and glucose-stimulated insulin secretion, as well as insulin content, reversed the increase in PEPCK mRNA expression and decrease in glycogen content in liver tissues of diabetic mice, and possessed strong anti-inflammatory and antioxidant activities	Zhou et al. (2015)
	*Anoectochilus roxburghii* (Wall.) Lindl	ARPs-p	Glucose (97.75%), galactose (1.20%), rhamnose (0.5%), arabinose (0.26%), xylose (0.23%) and mannose (0.06%)	97.283	β-(1–3)-linkedglucan, with only 2% of its backbone substituted at O-6 by→6)-β-D-Glcp-(1→ units and non-reducing end units of glucose	Mediates antioxidant activity, protects pancreatic islets from free radical damage, and ameliorates STZ-induced hyperglycemia and oxidative stress	Liu et al. (2020)
	*Tetrastigma h-emsleyanum* Diels et Gilg	THDP-3	Rhamnose, arabinose, mannose, glucose and galactose at molar ratio of 1.0:1.3:2.5:2.3:3.1	77.98	→4)-α-D-GalAp-(1→,→4)-β-D-Galp-(1→ and →4)-α-D-Glcp-(1→, and main branches of β-D-Manp-(1→,→3,6-β-D-Manp-1→ and α-D-Araf-(1→	Regulates key hepatic glycogen metabolism-related enzymes to improve glycogen synthesis and breakdown for effective glycogen control	Ru et al. (2019)
	*Phaseolus vul-garis* L	CIE2-F	1.00:0.56:1.29:0.88:64.38:1.30:19.53:141.68:0.24:42.02 for Man:GlcN:Rha:GlcA:GalA:Glc:Gal:Xyl:Ara:Fuc	925	→4)-α-D-GalpA-(1→and→4)-α-D-GalpA-6-OMe-(1→residues, and the branches of CIE2F-F were linked at the O-3 of →3,4)-R1 → 2)-α-D-Xylp-(1→ or R1 → 2)-α-D-Xylp-(1 → 2)-α-D-Xylp- (1→ (R1:α-L-Fucp-(1→, β-D-Xylp-(1→, β-D-Galp-(1→)	Promotes the restoration of intestinal microbial balance by altering the intestinal flora of Desmodium, *Bacillus*, Dantoin and *Actinomyces*	Qu et al. (2024)
	*Angelica sine-nsis* (Oliv.) Diels	APS-1I	Rhamnose (Rha), glucose (Glc), galactose (Gal), and arabinose (Ara) in a ratio of 1.0:6.9:4.1:2.3	17	α-1,6-Glcp, α-1,3,6-Glcp, α-1,2-Glcp, α-1,4-Galp, α-1,3- Rhap, α-1,3,5-Araf, α-1,3-Araf,α-1,4-Galp, β-1,3-Galp, β-1,4-Glcp	Improvement of IR and reversal of hepatic RAGE-JNK/p38-IRS signaling pathway	Liu et al. (2022)
		APS-2II	Glc	10	α-1,6-Glcp, α-1,3-Glcp, α-1,2-Glcp, and α-T-Glcp		
	*Lactaruis vole-mus* Fr	LV-P4-1	Fuc, Gal, Glc, Man, and GlcA	5.89	------	Influencing the structure of the gut microbiota	Wang et al. (2025)
	*Pachyrrhizus erosus*	PEP	Mannose: rhamnose: glucosamine: glucose: galactose: xylose: arabinose was 5.4:1.7:8.5:160.7:11.8:1:2.7	11.4	------	Regulates serum levels of glycated serum proteins, total TG and TC and protects the tissue structure of the pancreas, liver and kidneys	Ma et al. (2018)
	*Sarcandra gla-bra* (Thunb.) Nakai	SERP1	Fucose (Fuc), rhamnose (Rha), arabinose (Ara), galactose (Gal), glucose (Glc), mannose (Man), xylose (Xyl), galacturonic acid (GalA) and glucuronic acid (GlcA), with the molar ratio of 3.30:8.75: 11.82: 19.41: 8.77: 1.00: 10.77: 72.61: 27.51	42.08	1,4-linked α-d-galacturonic acid, methyl esterified 1,4-linked α-d-galacturonic acid, 1,4-linked α-d-glucuronic acid, 1,5-linked α-l-arabinose, 1,3-linked β-d-galactose 1,4-linked α-d-glucose, 1,4,6-linked β-d-glucose, 1,6-linked β-d-glucose, and 1,2-linked rhamnose	Increases glucose utilization in peripheral hepatic tissues and inhibits hepatic injury	Liu et al. (2017a)
	*Dendrobium o-fficinale* (Kimura & Migo)	DOP-1	Mannose and glucose at a molar ratio of 5.18:1 and 4.78:1	6.8	→4)-β-D-Glcp-(1→,→4)-β-D-Manp-(1→, →4)-2-O-acetyl-β-DManp-(1→ and →4)-3-O-acetyl-β-D-Manp-(1→	Reduces FBG levels by stimulating GLP-1 secretion	Kuang et al. (2020)
		DOP-2		14.3			
	*Medicago sati-va* L	MSP-II-a	Arabinose, glucose, galactose, mannose, rhamnose, and xylose in a molar ratio of 2.1 : 4.0 : 1.1:0.4 : 1.4 : 1.1	43	1,4-p-Glc, 1,3,4-Rha, and 1,3-p-Gal glycosidic linkages, revealing a mesh-like texture with irregular blade shapes	Reduced FBG levels and increased hepatic glycogen synthesis significantly alleviated IR.	Ma et al. (2024)
Marine algae	*Ulva prolifera*	EP	Rhamnose, glucose, glucuronic acid, xylose, galactose, arabinose, and mannose, and the molar ratio of each monosaccharide was 39.3, 23.4, 13.6, 12.5, 8.9, 1.4, and 0.9%	6.625	------	Enhancement of AKT phosphorylation, which promotes phosphorylation and resulting inactivation of downstream target GSK and enhances hepatic glycogen synthesis	Lin et al. (2024)
	*Ulva lactuca* L	ULP-1	Man, Rha, GluUA, Glu, Gal, Ara, and Xyl at a molar ratio of 0.22:22.88:9.41:0.44:0.50:3.44:0.60. Notably, Rha and GluUA.	42.91	β-D-Xylp-(1→3)-β-D-Arap-(1→6)-β-D-Galp- (1→6)-β-D-Glcp linked with [→α-L-Rhap-(1→4)-β-D-GlcpA→]n and α-DManp-(1→4)-α-L-Rhap (2SO3−)-(1→2)-α-L-Rhap (4SO3−)-(1→2)-α-L-Arap-(1→2)-α-L-Rhap-(1→ as its side chains at β-D-Glcp	Increases the abundance of relevant beneficial genera in the intestinal flora, reduces inflammation and lowers blood glucose values by modulating the levels of SCFAs	Chen et al. (2022b)
Fungi	*Naematelia a-urantialba*	NAP-3	Dmannose, D-rhamnose, and D-xylose with a molar ratio of 67.39: 7.87: 22.91	428	β-1, 3-D-Manp, β-1, 2, 3-D-Manp, β-D-Xylp, β-1, 4-D-Glcp, β-1, 4-D-Rhap in a molar ratio of 6.49: 1.11: 2.4: 0.13: 0.83	Increased insulin tolerance and increased anti-oxidative stress levels	Sun et al. (2023a)
	*Cordyceps mil-itaris* (L.ex Fr.) Link	EPS-III	D-Mannose, D-Glucose, and D-Galactose with mole ratio of 1.68 : 1: 1.83	1.56	→4)-α-D-Galp-(1→, while →3, 6)-α-D-Manp-(1→, →4)-α-D-Manp-(1→, →3)-β-D-Galp-(1→ and →3)-α-D-Glcp-(1→ were distributed in the backbone or in the branch chains	Relieves weight loss, lowers blood glucose concentration, improves glucose tolerance, protects immune organs and repairs dyslipidemia	Sun et al. (2021)
	an endophytic fungus polysaccharide FP from *Dendrobium officinale* Kimura & Migo	FP	Glucose, galactose, and mannose at a molar ratio of 2.1:3.4:3.9	2.53	→3,6)-β-L-Man-(1→, α-D-Glc-(1→, →4)-α-D-Glc-(1→, →3,6)-β-D-Gal-(1→, and →6)-β-D-Gal-(1→	Inhibits glycation at its early, intermediate, and late stages, blocking the formation of intermediate dicarbonyl compounds and late-stage advanced glycation end products (AGEs)	Zeng et al. (2019)
	*Catathelasma ventricosum*	CVP-1S	Glucose (94.2%), galactose (3.51%) and fucose (1.3%)	15	(1→6)-β-d-Glcp glycosidic bonds, and branches are attached to the backbone through 1,3-linked glycosidic bonds	Antioxidant, hypoglycemic and hypolipidemic activities	Liu et al. (2016)
	*Grifola frond-osa*	GFP-N	L-arabinose, D-mannose and D-glucose	1,260	→2,6)-α-D-Manp-(1 → 4, α-L-Araf-C1→, and →3,6)-β-D-Glcp-(1 →	Improve oral glucose tolerance and reduce IR.	Chen et al. (2019)
	*Lactaruis volemus* Fr	LV-P4-1	Fucose (Fuc), rhamnose (Rha), galactose (Gal), glucose (Glc), xylose (Xyl), mannose (Man), and glucuronic acid (GlcA) in a concentration ratio of 6.956:0.623:13.418:24.230: 0.622:5.298:1.088	5.89	t-Fuc(p), t-Man(p), t-Glc (p), t-Gal(p), 3-Man(p), 3-Glc(p), 2-Man(p), 2-Glc(p), 4-Gal(p), 6-Glc(p), 6-Gal(p), 3,6-Glc(p), 2,6-Man(p) and 2,6-Glc(p)	Influencing the structure of the gut microbiota and modulating the metabolic profile by altering the activity of certain metabolites to alleviate hyperglycemia	Wang et al. (2025)

### 3.1 Molecular weight

The hypoglycemic efficacy of polysaccharides is significantly modulated by their molecular weight, a pivotal structural determinant governing biological activity. Polysaccharides must have a molecular weight within an appropriate range to exhibit optimal activity. This is because a larger molecular weight results in a larger molecular volume, increasing transmembrane resistance and impairing absorption and utilization, thereby affecting hypoglycemic activity. Conversely, polysaccharides cannot form active structures if their relative molecular mass is too low, thereby reducing their hypoglycemic activity (Yulu et al., 2004; Gong et al., 2017)^.^

Studies have shown that prepared three Chinese yam polysaccharides (HSY, huaishanyao in Chinese) with different molecular weights, namely HSY-I (>50 kDa), HSY-II (10–50 kDa), and HSY-III (<10 kDa). After administering these three yam polysaccharides to T2DM mice, it was found that HSY-I and HSY-II exhibited significant hypoglycemic effects, while HSY-III, although also having hypoglycemic effects, also affected the FBG levels of normal mice, indicating that polysaccharides with too low molecular weight may have impaired hypoglycemic activity (Li Q. et al., 2017). Morever some researchers compared the hypoglycemic effects of *Pseudostellaria heterophylla* (Miq.) Pax polysaccharide (PHP) with molecular weight ranges of 7–210 kDa, 50–210 kDa, and 10–210 kDa on T2DM rats (Hu et al., 2013). The results showed that PHP with a molecular weight distribution of 50–210 kDa exhibited significant hypoglycemic effects, improved IR, enhanced anti-IR levels, and improved lipid metabolism disorders in rats with T2DM, making it a promising candidate drug for the treatment of T2DM.

### 3.2 Monosaccharide composition

As research has progressed, it has been discovered that most polysaccharides with hypoglycemic activity possess 1→3, 1→4, and 1→6 glycosidic bonds.

The latest research found that compared to crude *Armeniaca sibirica* L. Lam polysaccharide, AP-1, a neutral polysaccharide with a triple-helix structure extracted from Armeniaca sibirica L. Lam., exhibited stronger α-glucosidase inhibitory activity and free radical scavenging capacity (Peng et al., 2023). Consequently, AP-1 emerges as a promising natural candidate for diabetes intervention, functioning both as a glucose-regulating agent and an oxidative stress antagonist (Peng et al., 2023). Liu et al. (2016) found that the skeleton of *Catathelasma ventricosum* (Peck) Singer polysaccharide (CVP-1S) is primarily composed of (1→6)-β-d-Glcp glycosidic bonds, with branches connected to the skeleton via 1,3-linked glycosidic bonds, exhibiting antioxidant, hypoglycemic, and hypolipidemic activities. Researchers have discovered that purified *Hovenia dulcis* Thunb. polysaccharides (HDPs-2A) extracted from Hovenia dulcis can improve abnormal symptoms such as hyperglycemia and hyperlipidemia in T2DM rats, enhance insulin secretion, and thereby alleviate T2DM (Yang et al., 2022). The chemical structure of HDPs-2A is →5)-α-L-Araf-(1→, →5)-α-L-Araf-(1→, →3,5)-α-L-Araf- (1→, →6)-β-D-Galp-(1→, →3,6)-β-D-Galp- (1→, T-β-D-Galp, →3)-β-DGalp-(1→, and T-α-D-Glcp. It contains 1→3 and 1→6 glycosidic bonds, and its hypoglycemic activity is considered to be related to its branched structure. Liu et al. (2020) purified *Anoectochilus roxburghii* (Wall.) Lindl. polysaccharide (ARPs-p) from Anoectochilus roxburghii, which exhibits significant anti-diabetic activity, mediates antioxidant activity, protects pancreatic islets from free radical damage, and improves hyperglycemia, oxidative stress, and hyperlipidemia in STZ-induced T2DM mice. These findings constitute the inaugural evidence delineating how the principal bioactive constituent of ARPs-p specifically β-(1→3)-D-glucan modulates blood glucose regulation, thereby establishing a foundational framework for elucidating polysaccharide structure-efficacy correlations. Researchers extracted and purified a novel polysaccharide (THDP-3) from *Tetrastigma hemsleyanum* Diels et Gilg, THDP-3 consists of →4)-α-D-GalAp-(1→, →4)-β-D-Galp-(1→ and →4)-α-D-Glcp-(1→, and main branches of β-D-Manp-(1→, →3,6-β-D-Manp-1→ and α-D-Araf- (1→ chains. THDP-3 exerts its hypoglycemic effect by promoting glycogen synthesis and inhibiting glycogenolysis, with its mechanism of action potentially involving two pathways: G6pase and AMPK (Ru et al., 2019). The hypoglycemic activity may be associated with the presence of 1→3, 1→4, and 1→6 glycosidic bonds. Therefore, THDP-3 may be a potential natural functional food for the prevention and relief of high blood sugar.

### 3.3 Branched structure

The type and number of side chains have a certain impact on the therapeutic effect of polysaccharides for T2DM. The more side chains there are, the better the therapeutic effect of the polysaccharides, and the therapeutic effect is also correlate positively with the type of side chains.

Studies have shown that (Liu et al., 2022) two novel homogeneous polysaccharides, APS-1I and APS-2II, derived from *Angelica sinensis* (Oliv.) Diels, can bind to RAGE, enhance IR, and revolve the RAGE-JNK/p38-IRS signaling pathway in the livers of rats with diabetes induced by a high-fat diet and STZ, thereby exerting a hypoglycemic effect. An arabinoglucan (APS-1d) isolated from Angelica sinensis can also bind to RAGE and has a protective effect on diabetic nephropathy. However, APS-1I has a higher affinity for RAGE than APS-1d. Structural characterization suggests that the affinity for RAGE is positively correlated with the number of branch types. APS-1d contains one type of branched chain, namely →4-β-Glcp-1→4-α-Galp-1→, while APS-2 II is a linear glucan with one type of branched chain, namely →3-β-Galp-1→3,5-α-Araf-1→3-α-Araf-1→. APS-1d contains one type, α-T-Araf-1→6-α-Glcp-1→6-α-Glcp-1→, while APS-2 II is a linear glucan. The more branch types present, the higher the affinity for glucose. Each of the three bioactive polysaccharides incorporates α-1,6-Glcp residues, with APS-2 II demonstrating the highest concentration at 88.40%. In contrast, APS-1d and APS-1 I exhibit comparable levels (32.44% and 37.55%, respectively), both markedly reduced relative to APS-2 II. This suggests that a certain amount of α-1,6-Glcp may be essential for the interaction between polysaccharides and RAGE.

### 3.4 Hydrogen bond

Recent research have (Chen Y. et al., 2022) shown that *Ulva lactuca* L. polysaccharide (ULP-1) can enhance the genus ratio of the gut microbiota by regulating the abundance of SCFAs, thereby alleviating inflammation, lowering blood glucose levels, and alleviating T2DM. The structure of ULP-1 also forms strong interactions with target proteins through hydrogen bonds and van der Waals forces, particularly with GLP-1 (−10.34 kcal/mol), p16Ink4a (−10.51 kcal/mol), and GLP-1R (−8.57 kcal/mol). Additionally, the average hydrogen bond length is 2.36 MPa, which is shorter than the average length of traditional hydrogen bonds. This may be the reason behind ULP-1’s potential for treating T2DM.

In summary, within the current scientific research framework, “structure determines function” stands as the universally acknowledged core principle within academia. The molecular structure of polysaccharides holds the key to unraveling their mechanism of action against T2DM. It must be clarified that the structure-activity relationship governing polysaccharide anti-T2DM effects is not determined by any single structural factor in isolation but rather arises from the synergistic interplay of their primary structure, higher-order structure, and physicochemical properties. Existing research findings indicate that polysaccharides exhibiting the concurrent characteristics of “α β-(1→3)-glycosidically linked glucan backbone, a medium molecular weight of 10^4^–10^6^ Da, a triple-helix secondary structure, and good water solubility” typically maximize their anti-T2DM activity, demonstrating optimal glucose metabolism regulation effects.

## 4 Pathogenesis of T2DM

### 4.1 Insulin resistance

The synthesis, release, and action of insulin must be precisely regulated to meet the metabolic demands of the body. The biological action of insulin is exerted through binding to specific receptors on the cell membranes of target tissues, including skeletal muscle, adipose tissue, liver, and kidney, thereby initiating the phosphorylation of receptor substrates. Disruption of insulin receptor binding or signaling pathways impairs the proper expression of insulin’s biological effects, leading to the development of IR (Shu et al., 2021; Turner et al., 2017). In the context of IR, pancreatic β-cell function becomes compromised, resulting in abnormal insulin secretion and impaired glucose metabolism. Although compensatory insulin secretion increases, it is insufficient to effectively lower blood glucose levels or may even fail to prevent hyperglycemia. This prolonged imbalance between insulin secretion and glucose utilization further exacerbates pancreatic β-cell dysfunction (Cho et al., 2018), ultimately contributing to the pathogenesis of DM. The mechanisms underlying IR are multifactorial. Researchers demonstrated that the PI3K-Akt/PKB signaling pathway, which is activated by insulin, plays a crucial role in this process (Liu H. et al., 2019). Akt/PKB regulates numerous downstream effectors, including the translocation of glucose transporter proteins to the cell membrane, thereby facilitating glucose uptake. Consequently, any impairment in the Akt/PKB pathway or its downstream signaling molecules may result in IR (Liu H. et al., 2019). Furthermore, research has found that lipotoxicity, mediated by the accumulation of lipid intermediates such as diacylglycerol (DAG), ceramides, and TG, induces cellular stress and promotes the development of IR (Shulman and Petersen, 2018). IR is a critical factor in the pathogenesis of T2DM. Chronic disruption of the insulin secretion-glucose utilization balance, coupled with dysregulation of the PI3K-Akt/PKB pathway, significantly contributes to the progression of IR. Therefore, therapeutic strategies targeting the alleviation of IR represent a promising approach for the management of T2DM.

### 4.2 Inflammations

Inflammation represents the body’s overall physiological response to foreign substances, including disease-causing organisms, speck of dust, and viruses. Based on distinct inflammatory processes and cellular mechanisms, inflammation is primarily classified into acute and chronic forms. The latest research suggests that inflammatory responses are important contributing factors to chronic diseases, including diabetes, cardiovascular disease, autoimmune diseases, cancer, obesity, eye diseases, and digestive system diseases (Arulselvan et al., 2016). Recent research has demonstrated that Interleukin-17(IL-17)-mediated inflammation significantly contributes to the pathogenesis of numerous autoimmune and inflammatory diseases (Velikova et al., 2021). Specifically, IL-17-mediated inflammation has been implicated in the development of T2DM, with low-grade systemic inflammation identified as a key causative factor in T2DM pathogenesis, as evidenced by the studies of Velikova et al. (2021) and Hamid Akash et al. (2013). Among different inflammatory mediators, Interleukin-6(IL-6) exerts significant effects on glucose metabolism and homeostasis across multiple tissues and cell types, including peripheral tissues, adipocytes, neuroendocrine cells, and pancreatic islets, through direct or indirect mechanisms. Consequently, elevated IL-6 levels are closely related to the onset and development of T2DM (Lehrskov and Christensen, 2019). Furthermore, studies have revealed that the suppressor of cytokine signaling-3 (SOCS-3) may act as an inhibitor of insulin signaling. The pro-inflammatory cytokine IL-6 can exacerbate IR by inducing SOCS-3 overexpression (Gruber et al., 2014; Yogalakshmi et al., 2014). Additionally, IL-6 impairs the phosphorylation of insulin receptors and insulin receptor substrate-1(IRS-1), while IL-17 overexpression reduces insulin sensitivity, further contributing to IR through the activation of pro-inflammatory signaling pathways. These mechanisms collectively promote the pathogenesis of T2DM (Rehman et al., 2017; Tsai et al., 2015). Moreover, research has shown that levels of pro-inflammatory cytokines, including tumor necrosis factor-α (TNF-α) and interleukin-1β (IL-1β), are significantly elevated in T2DM patients compared to healthy individuals. The expression of these cytokines increases with disease progression (Wu et al., 2015). Researchers have observed that T2DM mice exhibited significantly higher expression of TNF-α, nuclear factor-κBp65 (NF-κBp65), and other related inflammatory factors compared to normal mice (Chen and Meng, 2022). These inflammatory factors ultimately induce inflammatory damage to pancreatic islet cells, leading to β-cell dysfunction and the development of T2DM. These findings underscore the critical role of inflammation in T2DM pathogenesis.

**FIGURE 2:**
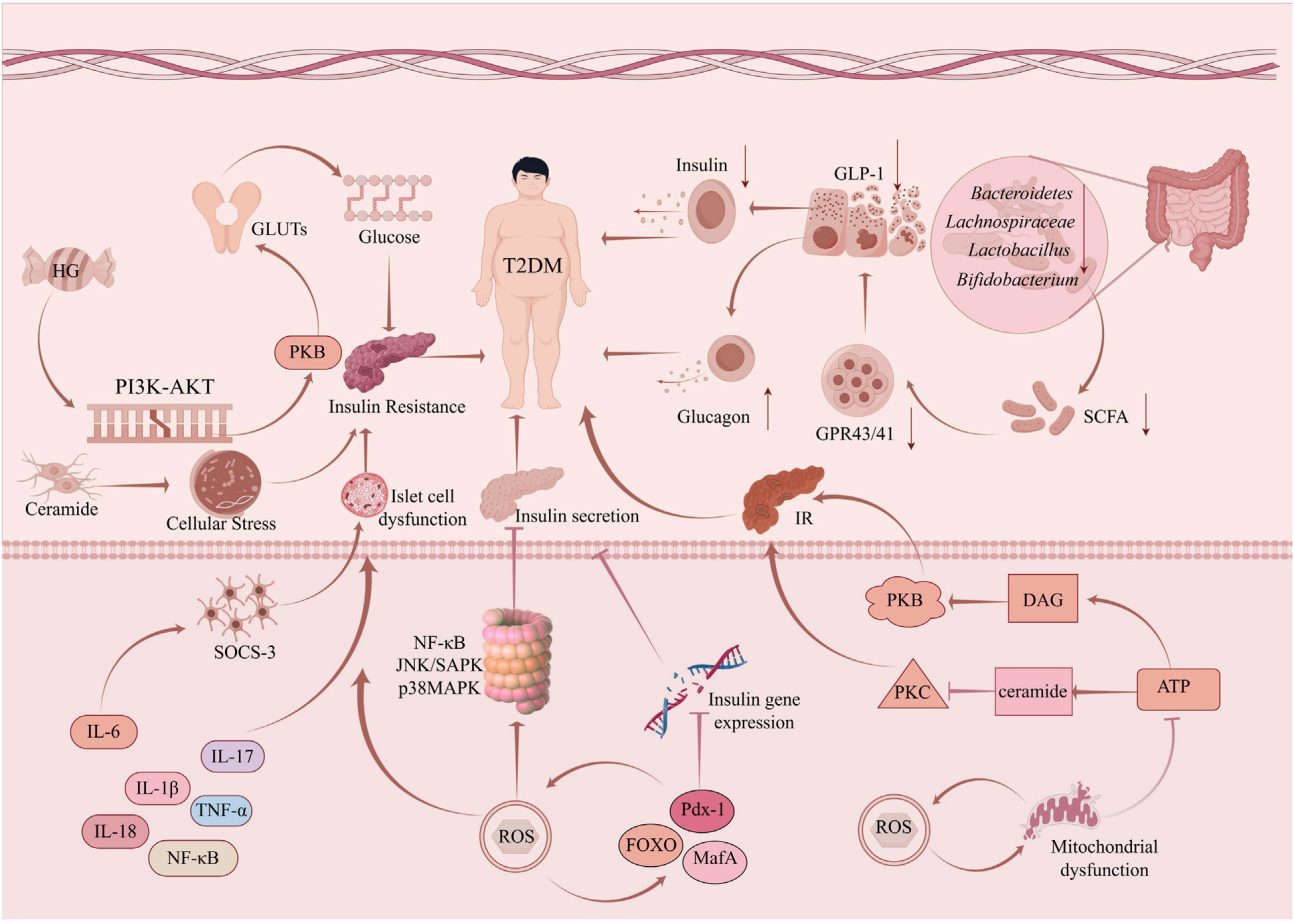
Core pathogenic mechanisms of T2DM. This schematic summarizes the key interconnected pathophysiological pathways driving T2DM development and progression. The central processes include: (1) Insulin Resistance (IR): Impaired PI3K-AKT signaling and glucose transporter (GLUTs) function, alongside lipotoxicity from ceramide and DAG accumulation, which activate stress kinases (PKC, JNK) and disrupt insulin action. (2) Inflammation: Elevated pro-inflammatory cytokines (e.g., IL-6, TNF-α) activate pathways like NF-κB, JNK/SAPK, and p38MAPK, promoting SOCS-3 expression which further inhibits insulin signaling. (3) Intestinal Gut Microbiota Dysbiosis: An imbalance in key bacterial groups (e.g., reduced *Bacteroidetes*, *Lachnospiraceae;* potential increases in *specific Lactobacillus* spp.) leads to altered Short-Chain Fatty Acid (SCFA) production. This affects GLP-1 secretion via GPR43/41 receptors, crucial for insulin and glucagon regulation. (4) Oxidative Stress: Excessive Reactive Oxygen Species (ROS) damage pancreatic β-cells, inhibiting key transcription factors (PDX-1, FOXO, MafA) essential for insulin gene expression, leading to islet cell dysfunction and impaired insulin secretion. (5) Mitochondrial Dysfunction: Defects in energy (ATP) production and metabolism exacerbate oxidative stress and contribute to IR, forming a vicious cycle that perpetuates β-cell failure and hyperglycemia (Depicted by Figdraw).

Collective evidence establishes inflammation as a primary pathogenic driver underpinning DM progression and the emergence of associated chronic disorders. The elevation of inflammatory factors such as NF-κBp65, TNF-a, IL-1β, IL-18, and IL-6, concurrent with activation of associated pro-inflammatory signaling pathways, disrupts glucose metabolism and homeostasis, damages insulin receptors, reduces insulin sensitivity, and causes pancreatic islet cell injury. These events culminate in pancreatic β-cell dysfunction, ultimately leading to the development and progression of T2DM (Figure 2).

### 4.3 Intestinal gut microbiota

The human gut is colonized by a vast array of bacteria that have co-evolved with the host. An individual’s gastrointestinal tract harbors a diverse community of approximately 300–500 distinct bacterial species. The colon, in particular, sustains a highly complex and dynamic ecosystem with an exceptionally high microbial density, where bacterial concentrations can reach 10^11^ to 10^12^ cells per gram of content (Francisco and Malagelada, 2003). The normal gut microbiota is primarily dominated by anaerobic bacteria, which are classified into six major phyla: *Verrucomicrobia*, *Fusobacteria*, *Proteobacteria*, *Actinobacteriota*, *Bacteroidetes*, and *Firmicutes* (Ma et al., 2019). Researchers conducted a comparative analysis of gut microbiota between T2DM mice (TSOD) and non-diabetic mice (TSNO) (Horie et al., 2017). Their findings revealed that the abundance of *Lactobacillus* spp. was significantly higher in TSOD mice compared to TSNO mice. Conversely, TSNO mice exhibited higher proportions of *Bacteroidetes* and *Lachnospiraceae*. Notably, certain genera, such as *Turicibacter and Clostridium* spp., were exclusively detected in the intestines of TSOD mice, suggesting a potential link to the metabolic abnormalities associated with T2DM. Wang Y. et al. (2020) demonstrated that SCFAs, produced by specific gut bacteria such as *Lactobacillus* and *Bifidobacterium*, play a crucial role in glucose metabolism. SCFAs upregulate the activity of G-protein-coupled receptors 43/41 (GPR43/41), which in turn enhances the secretion of GLP-1. GLP-1 modulates glucose-induced insulin secretion by enhancing β-cell functionality and sensitivity, thereby normalizing glucose homeostasis. Beyond its appetite-modulating effects, GLP-1 enhances glucose homeostasis through dual pancreatic mechanisms: activating insulin secretion in β-cells while suppressing glucagon output from α-cells, synergistically promoting metabolic control.

Distinct variations in microbial community profiles emerge when comparing individuals with T2DM to healthy controls, revealing fundamental shifts in gut ecosystem architecture tied to host metabolic status. For instance, the proportions of *Bacteroides* and *Trichoderma* differ markedly, with *Bacteroides* torsionis exclusively present in the intestines of T2DM mice. The SCFAs produced by *Lactobacillus* and *Bifidobacterium* promote GLP-1 secretion, enhancing β-cell function and glucose-stimulated insulin secretion, ultimately normalizing glucose homeostasis. These findings underscore the critical role of gut microbiota dysbiosis in the pathogenesis of T2DM.

### 4.4 Oxidative stress

Free radicals are physiologically essential components of biological homeostasis (Yaribeygi et al., 2019a; Yaribeygi et al., 2019b). However, when their output exceeds the antioxidant capacity of the organism, oxidative stress occurs. Oxidative stress is a critical contributor to the development of diabetic complications and IR (Yaribeygi et al., 2019a; Yaribeygi et al., 2018), and one of the key factors in the pathogenesis and progression of T2DM (Li G. et al., 2018). Pancreatic β-cells are particularly vulnerable to reactive oxygen species (ROS) due to their limited antioxidant defense mechanisms, making them prone to dysfunction and apoptosis under oxidative stress (Bigagli and Lodovici, 2019). Additionally, ROS can activate multiple stress-sensitive cellular pathways associated with IR and impaired insulin secretion (Bigagli and Lodovici, 2019). Researches have shown that oxidative stress induces pathways such as nuclear factor-κB (NF-κB), stress-activated protein kinase (JNK/SAPK), p38 mitogen-activated protein kinase (p38MAPK), and hexosamine, which collectively impair insulin secretion (Wronka et al., 2022). Furthermore, ROS can disrupt pancreatic β-cell development and interfere with insulin signaling pathways, thereby compromising insulin secretion and function (Macdonald Ighodaro, 2018; Yaribeygi et al., 2020). At elevated concentrations, free radicals exacerbate oxidative stress, which inhibits critical nuclear transcription factors involved in insulin gene expression. These factors include pancreatic duodenal homeobox-1 (PDX-1), forkhead box protein O (FOXO), and v-maf musculoaponeurotic fibrosarcoma oncogene homolog A (MafA). The inhibition of these transcription factors reduces β-cell proliferation and differentiation, ultimately decreasing insulin production at the DNA level (Yaribeygi et al., 2020).

These experimental findings elucidate how oxidative stress triggers activation of key signaling pathways including NF-κB, JNK/SAPK, p38MAPK and hexosamine cascades ultimately compromising pancreatic β-cell insulin secretory capacity. Simultaneously, excessive free radical concentrations inhibit critical transcription factors like PDX-1, FOXO, and MafA, which are essential for insulin gene expression. This inhibition reduces β-cell proliferation and differentiation, further diminishing insulin production. Collectively, these mechanisms contribute significantly to the development of T2DM.

### 4.5 Mitochondrial dysfunction

Mitochondria serve as the primary site for cellular ATP production and act as a metabolic hub for various biochemical reactions (Sreedhar et al., 2020). These compounds further modulate the intrinsic apoptosis pathway through governance of mitochondrial permeability transition pore opening and cytochrome C liberation, thereby orchestrating programmed cell death mechanisms (Kist and Vucic, 2021). The link between mitochondrial dysfunction and IR has been established through studies on obese and insulin-resistant individuals, who exhibit reduced mitochondrial oxidative capacity, impaired ATP synthesis, and defective lipid metabolism in skeletal muscle compared to insulin-sensitive populations (Kelley et al., 1999). Reduced mitochondrial biochemical activity, such as decreased mitochondrial numbers can lead to diminished oxidative protein content and activity (Kelley et al., 1999). This reduction in oxidative capacity results in the accumulation of lipids, including DAG and ceramides (Ivana et al., 2022). Both DAG and ceramides are known to inhibit insulin signaling. DAG inhibits insulin receptor activity by activating protein kinase C, which translocates to the plasma membrane (Samuel et al., 2010), while ceramides impair insulin signaling by inhibiting PKB or activating c-Jun N-terminal kinase, thereby contributing to IR (Bruce et al., 2012). These findings suggest that the accumulation of DAG and ceramides may serve as a potential mechanistic link between mitochondrial dysfunction and IR. Impaired energy metabolism, triggered by reduced mitochondrial biochemical activity, decreased mitochondrial content, and diminished oxidative protein function, is closely associated with the development of IR. This impairment disrupts redox homeostasis and metabolic adaptations, ultimately leading to metabolic disorders such as T2DM (Bruce et al., 2012). Additionally, abnormal mitochondrial autophagy, a selective process regulated by multiple signaling pathways, plays a critical role in mitochondrial dysfunction and the pathogenesis of T2DM. Mitophagy maintains intracellular homeostasis by removing damaged mitochondria, thereby making sure the mitochondria are working right (Bruce et al., 2012). The PINK1/Parkin pathway, which regulates mitochondrial fission and fusion, is essential for mitochondrial autophagy. This pathway not only facilitates the removal of damaged mitochondria but also modulates glucose metabolism and improves insulin sensitivity (Narendra and Youle, 2024; Fang X. et al., 2022). Conversely, impaired mitochondrial autophagy leads to the accumulation of damaged mitochondria, ROS and other toxic metabolites, which disrupt insulin signaling pathways and exacerbate IR (Scheibye-Knudsen et al., 2012; Yang et al., 2014).

The above research results indicate that the relationship between mitochondrial function and insulin signaling is highly complex. Mitochondrial dysfunction contributes to IR by disrupting energy metabolism, increasing oxidative stress, and impairing mitochondrial autophagy, all of which play significant roles in the development and progression of T2DM.

## 5 Therapeutic polysaccharides of natural origin improve T2DM by modulating related mechanisms

### 5.1 Regulating insulin resistance

IR is strongly associated with the onset of T2DM, and improving IR can significantly mitigate the progression of T2DM. Researchers have discovered that TNF-α impairs insulin-mediated tyrosine phosphorylation and PI3K-Akt activation, leading to reduced GLUT4 translocation and glucose uptake, thereby contributing to IR (Li J. et al., 2018). PI3K, an enzyme with serine/threonine protein kinase activity, plays a key role in the mechanisms of disease in T2DM. Additionally, insulin receptor substrate (IRS) serves as a critical mediator of insulin signaling and is essential for the regulation of post-receptor proteins in the insulin signaling pathway. Under the intervention of *Isatis tinctoria* polysaccharide (ITP), the expression of PI3K, IRS-1, GLUT4, and Akt was significantly upregulated, promoting glucose uptake and metabolism while enhancing insulin sensitivity. These findings suggest that ITP may alleviate IR through the PI3K-Akt pathway. Experimental results indicated that *Phellinus linteus* polysaccharide (PLPE) increased the abundance of SCFA-producing bacteria, thereby elevating SCFA levels (Liu Y. et al., 2019). This enhancement of SCFAs helps maintain intestinal barrier function, reduce blood lipopolysaccharide levels, and mitigate systemic inflammation, ultimately reversing IR. PI3K, a key kinase in skeletal muscle, mediates glucose entry into myofibers by regulating upstream factors of the insulin signaling pathway, thereby modulating GLUT4 translocation and improving insulin release, which alleviates T2DM (Widerska et al., 2018). Qiao et al. (2020) shown that *Schisandra chinensis* polysaccharide (SCAP) significantly suppressed the upregulation of phosphorylated c-Jun N-terminal kinase (P-JNK) by reducing levels of TNF-α, IL-6, IL-1β, C-reactive protein (CRP), and nuclear factor-kappa B (NF-κB). Simultaneously, SCAP increased the expression of phosphorylated IRS-1 (p-IRS-1), PI3K (p-PI3K), and p-AK), thereby improving insulin sensitivity. Similarly, MLP has been shown to upregulate the anti-apoptotic protein B-cell leukemia/lymphoma 2 (Bcl-2) while downregulating pro-apoptotic proteins Bcl2-associated X (Bax) and caspase-3 in pancreatic islet cells. MLP also reinstated the nuclear localization of pancreatic duodenal homeobox-1 (PDX-1) in diabetic rats, enhanced both the mRNA and protein expression levels of PDX-1 along with its downstream targets, GLUT2 and glucokinase (GCK), and provided protection to pancreatic islet cells against apoptosis. These effects improved the Homeostasis Model Assessment for IR (HOMA-IR), enhanced insulin secretion in pancreatic β-cells, and alleviated IR (Zhang et al., 2014). According to the latest in-depth research, *Ganoderma lucidum* polysaccharide (GLP) exhibits antidiabetic effects by reducing blood glucose levels, promoting hepatic glucose synthesis and storage, repairing damaged pancreatic islet cells, increasing insulin secretion, and reducing IR (Shao et al., 2022). Additionally, studies have shown that insulin-like growth factor 1 (IGF-1) synthesis in the liver promotes glucose uptake, and a decline in circulating IGF-1 levels is associated with increased IR, glucose intolerance, and T2DM (Sandhu et al., 2002; Succurro et al., 2009). Xinxin et al. (2023) treated HepG2 cells with APS in an IR model and found that APS enhanced insulin-like growth factor 1 (IGF-1) secretion by activating the signal transducer and activator of transcription 5 (STAT5) via the STAT5/IGF-1 signaling pathway.

**FIGURE 3:**
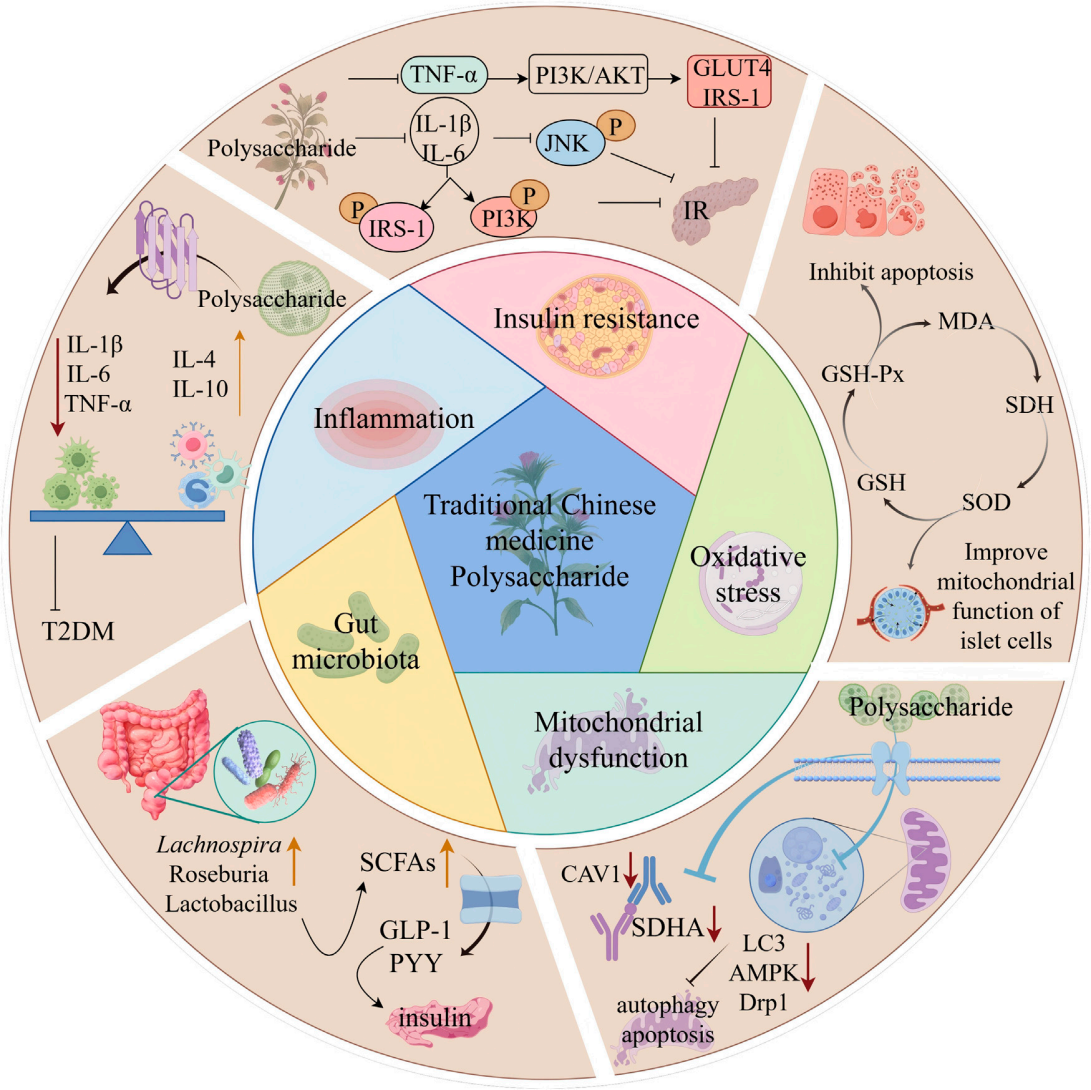
Multi-target mechanisms of natural polysaccharides in alleviating T2DM. This figure illustrates how natural polysaccharides from sources like traditional Chinese botanical drugs exert anti-diabetic effects by simultaneously targeting multiple pathological mechanisms: (1) Ameliorating Insulin Resistance: Polysaccharides enhance the insulin signaling pathway by upregulating key mediators like IRS-1 and PI3K, promoting glucose uptake. (2) Suppressing Inflammation: They reduce the levels of pro-inflammatory cytokines (IL-1β, IL-6, TNF-α) and modulate signaling to curb chronic inflammation. (3) Regulating Gut Microbiota: By modulating microbial composition, polysaccharides increase the production of beneficial metabolites like Short-Chain Fatty Acids (SCFAs), which in turn stimulate the secretion of gut hormones GLP-1 and PYY, improving insulin secretion and sensitivity. (4) Mitigating Oxidative Stress: Polysaccharides boost the activity of antioxidant enzymes (SOD, GSH-Px) and reduce markers of oxidative damage (MDA), thereby protecting pancreatic β-cells. (5) Improving Mitochondrial Dysfunction: They enhance mitochondrial function in islet cells by inhibiting excessive autophagy (via reducing AMPK, Drp1, LC3) and suppressing apoptosis (via the CAV1/SDHA pathway), leading to improved energy metabolism and cell survival. The integrated action across these pathways collectively inhibits apoptosis and restores glucose homeostasis (Depicted by Figdraw).

Numerous studies have demonstrated that various therapeutic polysaccharides of natural origin, including ITP, PLPE, and PRR, can effectively regulate glucose-lipid metabolism and ameliorate IR through modulation of key signaling pathways (PI3K/AKT/GLUT4 and STAT5/IGF-1) and regulation of associated protein expression (PI3K, IRS-1, GLUT4, IGF-1, and Akt). The multi-dimensional mechanisms through which polysaccharides alleviate T2DM are summarized in Figure 3 and Table 2.

**TABLE 2:** Mechanism of action of therapeutic polysaccharides of natural origin in modulating related pathways to improve T2DM.

Mechanisms	Types of therapeutic polysaccharides of natural origin	Model	Dosages	Passage	Expression of associated factors	Literatures
IR	ITP	3T3-L1 preadipocytes	25, 50, 100 ug·kg^−1^	PI3K-Akt	PI3K p85, IRS-1, GLUT-4, Akt↑	Li et al. (2018b)
	PLPE	T2DM rat	600 μg·kg^−1^	NF-κB/JNK	SCFAs↑ LPS↓	Liu et al. (2019b)
	SCAP	T2DM rat	25, 50, 100 mg·kg^−1^	PI3K/AKT	IL-1β, IL-6, TNF-α, CRP, NF-κB↓	Widerska et al. (2018)
	YPS	T2DM mouse	100, 200, 400 mg·kg^−1^	Proportion of intestinal flora communities, SCFA content	Thick-walled phylum to anamorphic phylum ratio, anamorphic phylum↓,*Lactobacillus*, the genus of bacteria↑, SCFAs↑	Qi et al. (2023)
	GLP	IR-HepG2 cell	0, 50, 100, 200, 400 μg·mL^−1^	P-AKT/AKT	IRS-1, P-AKT, P-GSK-3β, GLUT2↑, PEPCK↓	Shao et al. (2022)
	APC	IR-HepG2 cell		STAT5/IGF-1	IGF-1↑	Xinxin et al. (2023)
Inflammations	APS	Diabetic Nephropathy in rats	200, 400, 800 mg·kg^−1^	TLR4/NF-κB	IL-1β, IL-6, TNF-α↓, IκBα↑	Ren and Wang (2021)
	GPP	T2DM mouse	200, 400, 800 ug·mL^−1^	Levels of inflammatory factors such as IL-1β, IL-6, IL-4, and IL-10	MDA, TNF-α, IL-6 ↓, IL–4, IL-10, SOD↑	Wang et al. (2020b)
	SBP	T2DM mouse	50, 100, 200 mg·kg^−1^	NF-κB	ICAM-1, MCP-1↓	Honglai et al. (2023)
	LBP	T2DM rat	400 g·kg^−1^	Number of associated inflammatory factors	MDSCs↑, IL-17A, TNF-α, IL-6↓	Hai-Xia et al. (2020)
	SCP	T2DM rat	25, 50, 100 mg·kg^−1^	NF-κB	IL-6, CPR, NF-κB, TNF-α↓	Xingxu et al. (2020)
	SGP	T2DM mouse	50, 100, 200 mg·kg^−1^	TLR4-NF-κB	SOD↑, IL-6, TNF-α, MDA↓	Gong et al. (2021)
Gut flora	CCPP	T2DM rat	400 mg·kg^-1^	Proportion of acid-producing genera in the intestinal flora	GLP-1, PYY↑	Yao et al. (2020)
	CSP	T2DM rat	100, 200, 400 mg·kg^−1^	GPR41 signaling pathway	richoderma spp., Rochesteria spp., SCFAs, GLP-1, PYY↑	Jiang-xia et al. (2023)
	TMSP	T2DM mouse	100, 200, 300 mg·kg^−1^	Abundance of relevant probiotics in the intestinal flora	*Lactobacillus* spp., Rhodococcus spp., *Treponema* spp., Ruminalococcus spp.↑	Qiaoying et al. (2024)
	CMP	T2DM rat	1,000 mg·kg^−1^	Structure and relative abundance of intestinal flora	GLU, TC, LDL-C↓*,Bacteroidetes, Prevotella, Deltaproteobacteria, Oscillospira, Veillonellaceae, Phascolarctobacterium, Sutterella, and Bilophila*↑	Liu et al. (2018)
	GMP	T2DM Patient feces	—	Tryptophan-indolepyruvate metabolic pathway	Indoleacrylic acid, indoleacetic acid, indoleacetaldehyde, indolepropionic acid↑	Lanqi et al. (n.d.)
	PSP	T2DM mouse	10 mg·kg^-1^	Abundance of intestinal flora-associated genera	*Lactobacillus* spp.↓, Aspergillus, *Mycobacterium*, Verrucoccus, Escherichiaceae↑	Ming-chen et al. (2021)
Oxidative stress	PAC	T2DM rat	0.05, 0.1, 0.2 mg·kg^−1^	MAPK signaling pathway	GSH-Px, SOD↑, MDA↓	Li and Zhang (2022)
	AMP	T2DM mouse	—	—	GSH, SOD↑, MDA↓	Chen et al. (2022c)
	PSP	ARPE-19 cell	0, 6.25, 12.5, 25 mmol·L^−1^	oxidative stress products	ROS, MDA↓, SOD, GSH-Px↑	Wang et al. (2022b)
	MLP	T2DM rat	—	Quantity, activity of relevant oxidizing enzymes	MDA, SOD, CCO, SDH↑	Liu et al. (2017b)
Improvement of mitochondrial function	ASP	KK-Ay mouse	100, 200, 400 mg·kg^−1^	AMPKsignaling pathway	AMPK, Drp1, LC3↓	Jiang-xia et al. (2023)
	LNT	db/db mouse	10 mg·kg^−1^	CAV 1/SDHAsignaling pathway	CAV1, SDHA↓	Hu et al. (2023)

Polysaccharides modulate IR through multi-dimensional mechanisms, offering unique advantages in improving T2DM. They enhance insulin sensitivity by inhibiting the activity of enzymes involved in glucose metabolism, regulating gut microbiota, protecting pancreatic β-cells, and optimizing insulin signaling pathways. Among these, gut microbiota regulation is currently the most extensively studied pathway. However, the interaction between gut microbiota and polysaccharides represents a pivotal link. Given the inherent variability in gut microbiota composition across different populations, the regulatory effects of polysaccharides may exhibit individual differences. Consequently, future research should explore personalized intervention strategies based on individualized microbiota profiling.

### 5.2 Regulating inflammatory mechanisms

The inflammatory mechanism is a critical pathway contributing to the pathogenesis of T2DM, making its regulation essential for T2DM treatment. Recent research has found that APS, extracted from Astragalus membranaceus, as a highly effective bioactive compound (Ren and Wang, 2021). APS attenuated lipopolysaccharide (LPS)-induced inflammation by inhibiting the Toll-like receptor 4/nuclear factor-kappa B (TLR4/NF-κB) signaling pathway, reducing the expression of IL-1β, IL-6, and tumor necrosis TNF-α. This resulted in significant reductions in blood glucose and blood lipid levels in T2DM mice, improved IR, and alleviated T2DM symptoms. Additionally, APS demonstrated renoprotective effects in STZ-induced diabetic rats. Research has shown that *Gynostemma pentaphyllum* (Thunb.) Makino polysaccharide (GPP) reduced levels of alkaline phosphatase (ALP), alanine transaminase (ALT), aspartate transaminase (AST), and blood urea nitrogen (BUN) in T2DM mice (Wang Z. et al., 2020). GPP also enhanced the activities of antioxidant enzymes, including SOD, CAT, and GSH-Px, while decreasing MDA levels. Furthermore, GPP increased anti-inflammatory cytokines such as interleukin-4 (IL-4) and interleukin-10 (IL-10) and reduced pro-inflammatory cytokines like TNF-α and IL-6, thereby exerting hypoglycemic effects. Honglai et al. (2023) demonstrated that *Stachys baicalensis* Fisch. ex Bunge polysaccharides (SBP) inhibited the NF-κB signaling pathway and downregulated related factors, including intercellular adhesion molecule-1(ICAM-1) and monocyte chemoattractant protein-1 (MCP-1). This suppression of inflammatory responses reduced metabolic disorders, hepatic inflammatory injury, and hepatic fibrosis, alleviating T2DM symptoms. Hai-Xia et al. (2020) documented elevated myeloid-derived suppressor cell (MDSC) populations within the peripheral circulation and major lymphoid organs (spleen/liver) of diabetic rats following *Lycium barbarum* polysaccharide (LBP) administration. In parallel, LBP suppressed pro-inflammatory mediators TNF-α and IL-6, concurrently potentiating insulin receptor signaling pathways and augmenting β-cell secretory function collectively ameliorating T2DM pathogenesis. Researchers have discovered that *Schisandra chinensis* (Turcz.) Baill. polysaccharide (SCP) significantly reduced serum levels of pro-inflammatory factors, including IL-6, CRP, NF-κB, and TNF-α, in T2DM rats (Xingxu et al., 2020). By inhibiting systemic inflammation, SCP improved IR and repaired damaged pancreatic β-cells, contributing to blood glucose reduction. Similarly, Gong et al. (2021) demonstrated that *Siraitia grosvenorii* polysaccharide (SGP) downregulated the mRNA and protein expression of Toll-like receptor 4 (TLR4) and its downstream kinase NF-κB p65 in diabetic nephropathy (DN) mice. This inhibition of the TLR4-NF-κB pathway reduced inflammatory factors such as IL-6, TNF-α, and MDA while stimulating SOD production, thereby attenuating T2DM.

On the whole, therapeutic polysaccharides of natural origin such as APS, GPP, and LBP demonstrate significant potential in modulating inflammatory responses. They primarily alleviate systemic inflammatory states by inhibiting the TLR4–NF-κB signalling pathway, thereby reducing the expression and release of pro-inflammatory cytokines (e.g., IL-1β, IL-6, TNF-α) while elevating levels of anti-inflammatory factors. This series of anti-inflammatory actions not only aids in improving insulin resistance but also promotes functional repair and structural recovery of pancreatic β-cells. Consequently, natural polysaccharides exhibit multi-targeted hypoglycemic effects in the treatment of T2DM, demonstrating significant potential for clinical application.

### 5.3 Regulating intestinal flora

Maintaining a stable proportion of relevant genera in the intestinal flora iscrucial for blood glucose homeostasis, and modulating the gut microbiota playsa pivotal role in the treatment of T2DM. Studies have shown that *Cymbopogon schoenanthus* (L.) Spreng. polysaccharide (CCPP) increases the abundance of SCFA-producing bacteria in the intestines of T2DM rats, serving as a carbon source for SCFA production (Yao et al., 2020). SCFAs activate G protein-coupled receptors (GPCRs), promoting the secretion of GLP-1 and peptide tyrosine-tyrosine (PYY) by intestinal L-cells. This enhances insulin secretion, improves insulin sensitivity, reduces gluconeogenesis, and alleviates T2DM symptoms. Researchers have discovered that CSP treatment in T2DM rats increased the ratio of Firmicutes to Bacteroidetes and enhanced α-diversity (Li et al., 2020). The abundance of SCFA-producing genera, such as *Lachnospira* and *Roseburia*, increased with CSP intake, leading to elevated SCFA levels. SCFAs activate the GPR41 signaling pathway in intestinal endocrine cells, stimulating the secretion of GLP-1 and PYY.GLP-1 promotes insulin secretion from pancreatic β-cells, reduces glucagon levels, and regulates glucose absorption, thereby lowering blood glucose levels. Qiaoying et al. (2024) reported that *Trichosanthes rosthornii* Harms polysaccharide (TMSP) modulates the gut microbiota in T2DM mice. Elevated glucose levels disrupt the intestinal flora, reducing microbial abundance and diversity. TMSP treatment significantly increased the abundance of beneficial bacteria, suchas *Lactobacillus*, *Roseburia*, *Oscillospira*, and *Ruminococcu*s, restoring the gut microbiota to levels similar to those in healthy mice and effectively alleviating T2DM. At the same time, researchers have also discovered that CMP improves insulin tolerance, reduces serum glucose, TC, and LDL-C levels, and increases HDL levels in T2DM rats (Liu et al., 2018). CMP selectively enriches key bacterial species, including *Bacteroides*, *Proteus*, *Aspergillus*, *Treponema*, *Veronica*, *Caulobacter*, *Sartorius* and *Cholera* (Liu et al., 2018). These findings suggest that CMP’s therapeutic effects in T2DM are linked to the selective modulation of specific gut microbial communities, particularly the enrichment of SCFA-producing genera (Qi et al., 2021). In addition, researchers have found that *Glycine max* (L.) Merr. polysaccharides (GMP) significantly improve amino acid metabolism in the gut microbiota of T2DM patients (Lanqi et al., n.d.). GMP activates the tryptophan-indole pyruvic acid metabolic pathway, promoting the productionof indole metabolites such as indoleacrylic acid, indoleacetic acid, indoleacetaldehyde, and indolepropionic acid. These metabolites benefit intestinal health and blood glucose regulation, alleviating T2DM symptoms. Ming-chen et al. (2021) observed that PSP significantly increases the abundance and diversity of gut microbiota in T2DM mice. PSP treatment notably increased the relative abundance of *Lactobacillus* and *Bacteroidetes* while reducing the abundance of *Aspergillus*, *Anaplastic Bacteria*, *Wehrung’s Coccidioides*, *Escherichia*, and *Klebsiella*.

For the most part, therapeutic polysaccharides of natural origin such as CCPP, CSP, TMSP, CMP, GMP, and PSP improve T2DM by selectively modulating the gut microbiota. These polysaccharides enrich SCFA-producing bacteria, elevate SCFA levels, and activate the GPR41 signalling pathway, thereby enhancing GLP-1 and PYY secretion. This promotes insulin secretion, reduces glucagon levels, and regulates glucose absorption. Notably, therapeutic polysaccharides of natural origin modulate the gut microbiota through multiple pathways, including adjusting microbial composition, repairing the intestinal barrier, and regulating metabolic products. Polysaccharides can improve amino acid metabolism and increase the richness and diversity of the gut microbiota, thereby further alleviating T2DM. As previously noted, ‘structure determines function.’ Low molecular weight polysaccharides (e.g., <10 kDa) are typically more readily fermented and utilized by the microbiota, whereas high molecular weight polysaccharides (e.g., >1,000 kDa) may exert more sustained effects within the gut. Furthermore, polysaccharides containing acidic groups such as glucuronic acid generally exhibit more pronounced microbiota-modulating activity. It is worth considering that, as previously noted, responses to the same polysaccharide intervention may vary considerably due to individual differences in baseline gut microbiota composition. Furthermore, the lack of clarity regarding optimal dosing presents a significant challenge for the precise administration of polysaccharides.

### 5.4 Reducing oxidative stress

Oxidative stress is closely associated with the development and progressionof T2DM and its complications. Therefore, mitigating oxidative stress is crucial for alleviating T2DM and its associated conditions. According to recent reports, researchers demonstrated that *Wolfiporia cocos* (F.A. Wolf) Ryvarden & Gilb. polysaccharide (WCP) enhances the activity of antioxidant enzymes, including SOD, GSH-Px, and CAT, in the renal tissues of diabetic mice (Li and Zhang, 2022). These enzymes play a vital role in scavenging free radicals and protecting cells from oxidative damage. Additionally, WCP significantly reduced the levels of MDA, a lipid peroxidation product, in diabetic mice, indicating its ability to mitigate oxidative stress-induced cellular damage. These findings suggest that WCP protects cells from oxidative stress, thereby preserving their normal function and metabolic activity (Ao et al., 2022). Further studies have shown that APS inhibits high glucose-induced oxidative stress and apoptosis in mouse podocyte cells (MPC5) by upregulating the expression of silent information regulator 1 (SIRT1). This upregulation suppresses the extracellular signal-regulated kinase (ERK1/2) and p38MAPK signaling pathways, leading to reduced blood glucose and serum insulin levels in T2DM mice (Chen X. et al., 2022). Wang W. et al. (2022) constructed an *in vitro* diabetic retinopathy model and treated retinal pigment epithelial (RPE) cells with *Polygonum multiflorum* Thunb. polysaccharides (PMP). They found that PMP alleviated high glucose-induced oxidative stress in RPE cells by reducing intracellular ROS and MDA levels while increasing SOD and GSH-Px activities. These effects collectively mitigate the progression of diabetic retinopathy. Researchers discovered through experiments that MLP intervention in T2DM rats significantly decreased MDA content and enhanced the activities of SOD, mitochondrial cytochrome c oxidase (CCO), and succinate dehydrogenase (SDH) compared to the T2DM model group (Liu C-G. et al., 2017). These results indicate that MLP reduces oxidative stress injury, improves mitochondrial function in pancreatic islet cells, and protects pancreatic β-cells in T2DM rats.

Based on the above research, it can be concluded that, therapeutic polysaccharides of natural origin, such as WCP, APS, PMP, and MLP, enhance the activities of antioxidant enzymes (SOD, GSH-Px, CAT) and reduce oxidative stress markers (MDA). By scavenging free radicals and protecting pancreatic β-cells from oxidative damage, these polysaccharides maintain normal cellular function and metabolic activity, thereby alleviating T2DM and its complications.

### 5.5 Improving mitochondrial dysfunction

Increasing evidence indicates that mitochondrial dysfunction plays a key role in the development of T2DM, making the improvement of mitochondrial function a critical therapeutic target for T2DM (Li H. et al., 2017). For instance, mitochondrial autophagy-related proteins, including microtubule-associated protein 1 light chain 3 (LC3) and dynamin-related protein 1 (Drp1), are involved in the regulation of mitochondrial autophagy. Additionally, adenosine monophosphate-activated protein kinase (AMPK) is a key regulator of mitochondrial metabolism and autophagy (Iorio et al., 2021; Herzig and Shaw, 2018). Studies have shown that *Angelica* L. polysaccharide (ASP) downregulates the expression of AMPK, Drp1, LC3, and other related proteins, thereby attenuating mitochondrial autophagy by inhibiting the AMPK pathway and its downstream signaling molecules. This mechanism contributes to the alleviation of T2DM-related symptoms (Jiang-xia et al., 2023). Caveolin-1 (CAV1) interacts with succinate dehydrogenase subunit A (SDHA), a component of mitochondrial complex II, and plays a critical role in mitochondrial metabolism and autophagy. The binding of CAV1 to SDHA triggers the ubiquitination and degradation of SDHA, leading to mitochondrial dysfunction and apoptosis (Hu et al., 2023). However, treatment with Lentinan (LNT) inhibits this interaction, effectively improving mitochondrial function and reducing apoptosis (Hu et al., 2023).

Based on the aforementioned experimental findings, it is evident that thera-peutic polysaccharides of natural origin, such as ASP and LNT, can modulate mitochondrial function through multi-target regulation, offering a promising new therapeutic strategy for treating T2DM. On the one hand, they mitigate excessive mitochondrial autophagy by inhibiting the AMPK pathway and its downstream signaling, thereby maintaining mitochondrial homeostasis in pancreatic β-cells. On the other hand, they effectively suppress the mitochondrial apoptosis pathway by blocking the protein interaction between CAV-1 and SDHA. These actions collectively improve overall mitochondrial function, including enhancing energy metabolism, increasing ATP production, and reducing oxidative stress levels. This synergistically enhances insulin secretion capacity, ultimately mitigating the progression of T2DM.

## 6 Therapeutic polysaccharides of natural origin for the treatment of T2DM complications

**FIGURE 4:**
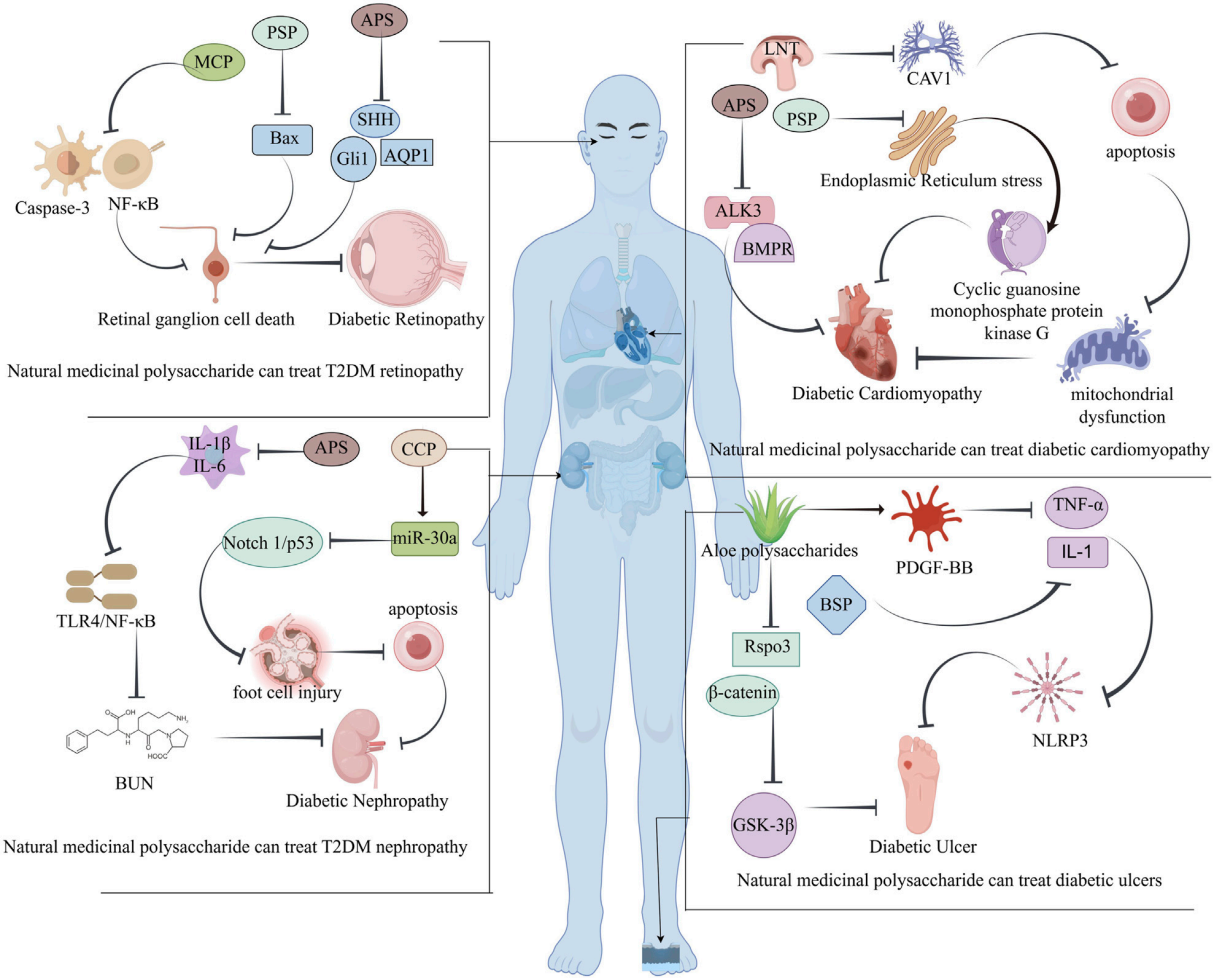
Schematic diagram of the mechanisms of therapeutic polysaccharides of natural origin in treating multiple complications of T2DM. This figure illustrates the specific action pathways of therapeutic polysaccharides of natural origin in treating four complications of T2DM. (1) For diabetic retinopathy, they regulate factors like MCP, PSP, and APS, and influence signaling molecules including Caspase-3, NF-κB, Bax, SHH, Gli1, and AQP1 to inhibit retinal ganglion cell death. (2) In diabetic cardiomyopathy, via factors such as LNT, APS, PSP, ALK3, and BMPR, they interfere with endoplasmic reticulum stress, the cyclic guanosine monophosphate protein kinase G pathway, and mitochondrial function, reducing cell apoptosis and improving myocardial injury. (3) For diabetic nephropathy, they modulate APS, CCP, IL-1β, IL-6, TLR4/NF-κB, Notch1/p53, and miR-30a to alleviate podocyte injury and cell apoptosis, while also affecting BUN levels to mitigate renal lesions. (4) In diabetic ulcers, by utilizing components like aloe polysaccharides, BSP, Rspo3, β-catenin, and GSK-3β, they regulate factors including PDGF-BB, TNF-α, IL-1, and NLRP3 to promote ulcer healing (Depicted by Figdraw).

T2DM is a prevalent chronic disease associated with severe vascular, renal, and neurological complications (Hauwanga et al., 2024; Conca et al., 2018). Although numerous treatments are available, many are accompanied by significant side effects. Therapeutic polysaccharides of natural origin, as the primary bioactive metabolites of botanical drugs, have demonstrated remarkable efficacy in treating diabetes and its complications with minimal toxicity or adverse effects DM (Zheng et al., 2019) (Figure 4).

### 6.1 Preventing diabetic retinopathy

Diabetic retinopathy (DR) is one of the most prevalent complications of Chronic hyperglycemia induces fragility and narrowing of retinal blood vessels, impairing blood and oxygen supply to the retina and ultimately leading to vision loss (Lin et al., 2021; Invernizzi et al., 2023). Research have shown that *Momordica charantia* L. polysaccharide (MCP) significantly alleviates retinopathy in diabetic rats by delaying retinal neurodegeneration and microvascular complications (Liu et al., 2023). MCP improved the anti-inflammatory and anti-apoptotic capacity of retinal tissues and emphasized the expression of NF-κB and Caspase-3 pathway proteins and genes, thereby inhibiting inflammation and apoptosis in retinal ganglion cells. Recently research have found that PSP intervention in diabetic rats reduced retinal vascular tortuosity and leakage, improved retinal ischemia, and reduced the expression of apoptosis-associated proteins, including Bax and p38 MAPK (Wang Y. et al., 2019). These effects collectively reduced retinal ganglion cell death, providing a therapeutic basis for PSP in treating diabetic retinal vascular diseases. Moreover, aquaporin 1 (AQP1) and Sonic Hedgehog (SHH) signaling pathways as critical regulators in DR pathogenesis (Qu et al., 2025). Glioma-associated oncogene homolog 1 (Gli1), the final effector of the SHH pathway, was also implicated (Qu et al., 2025). APS significantly attenuated the abnormal overexpression of AQP1, Gli1, and SHH in DR, improving retinal cell morphology in T2DM rats. These findings suggest that APS may treat DR by modulating the SHH-Gli1-AQP1 signaling pathway, offering a novel therapeutic approach for DR. Researchers reported that *Crassostrea gigas* polysaccharide (CGP) reduced random blood glucose and fasting insulin levels in T2DM mice (Chen et al., 2024). CGP also decreased vascular endothelial growth factor (VEGF) expression in retinal tissues, suppressed pathological angiogenesis, and improved retinal structural abnormalities and vascular network integrity, thereby slowing DR progression.

In summary, therapeutic polysaccharides of natural origin, such as MCP, PSP, APS, and CGP, mitigate DR by attenuating the overexpression of AQP1, Gli1, and SHH, downregulating NF-κB and Caspase-3 pathway proteins, and inhibiting retinal ganglion cell inflammation and apoptosis. Additionally, these polysaccharides delay retinal neurodegeneration and microvascular complications, reduce vascular tortuosity and leakage, improve retinal ischemia, and enhance the anti-inflammatory and anti-apoptotic capacity of retinal tissues, demonstrating significant therapeutic potential for DR.

### 6.2 Preventing and treating diabetic nephropathy

DN is a chronic flammatory complications of DM, characterized by hyperglycemia, proteinuria, and edema. It is one of the main causes of end-stage renal disease (ESRD) (Caihong et al., 2022). Studies have shown that APS effectively reduces FBG, BUN, and serum creatinine (Scr) levels, attenuates renal pathological damage, and inhibits the TLR4/NF-κB signaling pathway by downregulating inflammatory factors such as IL-1β, IL-6 and MCP-1. These effects significantly ameliorate renal damage in diabetic nephropathy rats (Guo et al., 2023). The miR-30 family plays a critical role in hyperglycemia and DN pathogenesis (Ren and Wang, 2021; Tang et al., 2019; Mao et al., 2018). Downregulation of miR-30a exacerbates podocyte injury in focal segmental glomerulosclerosis by activating the Notch1/p53 signaling pathway (Wu et al., 2014). Researchers have discovered that *Cordyceps cicadae* (Miq.) Lloyd polysaccharides (CCP) protect T2DM mice from inflammation and oxidative damage (Zheng et al., 2024). CCP upregulates miR-30a expression and its associated pathway proteins, reduces apoptosis, and improves renal function and morphology in DN mice. Wenting et al. (2023) found that *Zea mays* L. polysaccharides (ZMP) modulate the relative abundance of Firmicutes, Bacteroidetes, and Trichoderma-NK4A136 in the gut microbiota of DN mice. ZMP improves metabolic abnormalities of endogenous substances, such as glycerophospholipids, bile acids, aromatic amino acids, and uremic toxins, and significantly regulates the gut microbiota structure and endogenous metabolites, providing a basis for adjuvant treatment of T2DM and DN. Wan et al. (2022) demonstrated that Lycium barbarum polysaccharide (LBP) significantly reduces blood glucose levels and improves IR in T2DM mice. LBP also decreases Scr, BUN, and mRNA levels of tumor necrosis TNF-α, IL-1β, IL-6, and serum amyloid A3 (SAA3) in the renal cortex. Additionally, LBP reduces SAA3 protein deposition and circulating levels, mitigates glomerular and tubular injury, and exerts therapeutic effects on DN.

Based on the aforementioned research, it can be concluded that therapeutic polysaccharides of natural origin, such as APS, CCP, ZMP, and LBP, inhibit the TLR4/NF-κB signaling pathway, reduce inflammatory factor expression, and significantly improve renal damage in DN rats. These polysaccharides also upregulate miR-30a and its associated pathway proteins, reducing apoptosis and improving renal function. Furthermore, they effectively lower BUN and Scr levels, alleviate renal pathological damage, and improve renal morphology, highlighting their potential in the prevention and treatment of DN.

### 6.3 Preventing diabetic cardiomyopathy

Diabetic cardiomyopathy (DCM) is a key complication of DM, characterized by mitochondrial dysfunction and energy metabolism disorders. DCM patients often exhibit cardiac hypertrophy, cardiomyocyte hypoxia, and nutrient insufficiency, ultimately leading to heart failure and increased mortality (Sicheng et al., 2022; Algieri et al., 2022). Therefore, targeting mitochondrial dysfunction-related proteins holds significant clinical potential for DCM treatment. Studies have shown that Lentinan (LNT) inhibits the binding of CAV1 to SDHA, preventing SDHA ubiquitination and degradation. Silencing the CAV1 gene decreases apoptosis and enhances mitochondrial function, suggesting that LNT can be used as a potential treatment for DCM by mitigating mitochondrial dysfunction and apoptosis (Hu et al., 2023). Research has demonstrated that the bone morphogenetic protein 10 (BMP10)-mediated pathway is activated in high glucose (HG)-stimulated H9C2 cells and STZ-induced DCM rats (Sun S. et al., 2023). APS attenuated the increase in cardiomyocyte surface area, improved cardiomyocyte viability, enhanced cardiac function, and reduced the heart weight-to-body weight (HW/BW) ratio in DCM rats. APS also downregulated the expression of BMP10 and bone morphogenetic protein type II receptor (BMPRII), demonstrating significant anti-cardiac hypertrophic effects and therapeutic potential for DCM (Sun S. et al., 2023). At the same time, researchers also found that PSP improved cardiac function and alleviated high-fat diet-induced cardiac dysfunction in DCM mice (Lei et al., 2024). PSP protected diabetic myocardium by inhibiting endoplasmic reticulum stress, attenuating protein kinase R-like endoplasmic reticulum kinase (PERK) expression and oxidative stress, and enhancing the cyclic guanosine monophosphate (cGMP)-protein kinase G (PKG) signaling pathway, thereby ameliorating DCM.

Based on the above, it can be concluded that therapeutic polysaccharides of natural origin, such as LNT, APS, and PSP, alleviate cardiomyocyte hypertrophy, inhibit endoplasmic reticulum stress, improve cardiomyocyte viability, enhance cardiac function, and reduce the HW/BW ratio. These findings underscore the potential of natural polysaccharides in both the prevention and treatment of DCM, presenting a promising direction for future research endeavors.

### 6.4 Preventing and treating the diabetic foot

Diabetic foot (DF) is a severe chronic complication of DM, characterized by damage to the skin and deep tissues below the ankle joint, often accompanied by infections and/or lower extremity arterial occlusion, with severe involvement of muscle and bone tissues (Saseedharan et al., 2018). Diabetic foot ulcers (DFUs) and diabetic foot infections (DFIs) are hallmark pathological manifestations of DF (Mingyang et al., 2022). Other studies indicate that *Aloe vera* (L.) Burm.f. polysaccharide (AVP) increased the levels of basic fibroblast growth factor (bFGF) and platelet-derived growth factor-BB (PDGF-BB) while reducing serum inflammatory factors, including interleukin-1 (IL-1) and tumor necrosis TNF-α, in T2DM rats with DFUs compared to the DFU model group (Zheng et al., 2021). Additionally, AVP downregulated the expression of R-spondin 3 (Rspo3), β-catenin, and glycogen synthase kinase-3β (GSK-3β) proteins in wound tissues, demonstrating therapeutic potential for DFUs. Other experimental results have shown that *Bletilla striata* (Thunb.) Rchb.f. polysaccharide (BSP) accelerated wound healing, inhibited macrophage infiltration, and promoted angiogenesis in DFU model mice (Zhao, 2021). Biochemical analyses revealed that BSP reduced the levels of inflammatory factors IL-1β and TNF-α in the ulcer area. Furthermore, BSP suppressed the overactivation of NLRP3 inflammasomes and improved insulin sensitivity, suggesting that its wound-healing effects may be mediated through the inhibition of NLRP3 inflammasome activation in macrophages. Wang L. et al. (2020) reported that *Pseudostellaria heterophylla* (Miq.) Pax polysaccharide (PHP) accelerated wound scabbing and healing in DFU rats, demonstrating therapeutic efficacy. Additionally, studies showed that APS significantly reduced IL-1β levels in diabetic foot ulcer exudates and stimulated fibroblast proliferation, thereby accelerating DFU wound healing (Xiao et al., 2010).

Based on the above summary, it can be concluded that therapeutic polysaccharides of natural origin, such as AVP, BSP, PHP, and APS, promote the expression of bFGF and PDGF-BB, reduce serum inflammatory factors (e.g., IL-1 and TNF-α), and inhibit NLRP3 inflammasome overactivation. These mechanisms collectively accelerate wound healing and offer therapeutic benefits for DF.

## 7 Discussion and prospect

This comprehensive review systematically consolidates current understanding of how natural polysaccharides exert anti-type 2 diabetes mellitus (T2DM) effects through multi-target mechanisms. It provides a detailed analysis of polysaccharides derived from plants, algae, and fungi, elucidating the relationships between their structural diversity and the mechanisms underlying the alleviation of T2DM and its associated complications. A key innovative aspect of our analysis is the integration of structural features—such as molecular weight, monosaccharide composition, glycosidic linkages, and branching patterns—with their corresponding biological activities and mechanistic pathways. For instance, we highlight that specific structural motifs, including (1→3), (1→4), and (1→6) glycosidic bonds, are frequently associated with enhanced hypoglycemic and insulin-sensitizing effects, a correlation not sufficiently emphasized in earlier literature.

Furthermore, this review uniquely synthesizes evidence across diverse polysaccharide sources to reveal both shared and unique mechanisms of action. While many polysaccharides activate the PI3K/Akt pathway to improve insulin sensitivity, fungal-derived polysaccharides often exhibit additional immunomodulatory and mitochondrial protective effects, suggesting source-specific therapeutic advantages. By leveraging contemporary structural elucidation techniques, we correlate polysaccharide structures with their functional impacts on key pathogenic pathways of T2DM, including insulin resistance, inflammatory responses, oxidative stress, gut dysbiosis, and mitochondrial dysfunction. This holistic, mechanism-centered perspective not only bridges existing knowledge gaps but also provides a foundational framework for the rational, structure-based design of polysaccharide-based therapeutics for T2DM.

Despite the promising therapeutic potential of natural polysaccharides in managing T2DM, several significant limitations in current research must be acknowledged to guide future endeavors. A primary concern is the translational gap from preclinical models, as the majority of existing evidence is derived from cell lines or animal models, which may not fully recapitulate the complexity of human T2DM pathophysiology, thereby limiting the predictability of clinical efficacy and safety. This challenge is compounded by the inherent structural heterogeneity and standardization challenges of these macromolecules, where variations in molecular weight, branching, and substitution patterns pose substantial obstacles for quality control and batch-to-batch reproducibility, forming a major barrier to clinical translation. Furthermore, while the multi-targeted (polypharmacological) mechanisms are advantageous for a complex disease, they complicate the precise attribution of therapeutic effects to specific structural features or isolated pathways, hindering the development of precisely defined therapeutics. The clinical translation is further hampered by unclear pharmacokinetics and delivery hurdles, including poorly characterized *in vivo* metabolic fate, bioavailability, and optimal dosage windows, coupled with a lack of advanced formulation strategies for efficient and targeted delivery. Finally, there is an insufficient consideration of individualized responses, particularly for polysaccharides acting via gut microbiota modulation, as current research largely overlooks the impact of inter-individual variations in baseline microbiome composition and lacks strategies for personalized intervention.

To overcome these challenges and advance the field, future research must prioritize a multi-faceted approach. Advancing clinical translation through rigorously designed human trials (Phase I-IV) is imperative to validate efficacy, safety, and dosing in patients. Concurrently, deepening structural characterization and structure-activity relationship (SAR) studies is essential; this requires employing advanced technologies like high-resolution NMR, cryo-electron microscopy, and AI-assisted modeling to elucidate precise structure-function relationships for the rational design of improved therapeutics. Parallel efforts must focus on optimizing pharmacokinetic properties and drug delivery systems through targeted chemical modifications and the development of novel platforms (e.g., nanoparticles, hydrogels) to enhance bioavailability, stability, and targeting. Given the role of the gut microbiota, developing personalized intervention strategies based on individual microbial and metabolic profiles represents a critical frontier, necessitating large-scale cohort studies to understand how factors like diet and genetics influence response. A holistic understanding of their polypharmacology will require integrating multi-omics and systems biology approaches to map the complex, multi-layered interaction networks of polysaccharides within the host. The research scope should also be expanded by evaluating their potential in preventing and treating T2DM complications and assessing long-term outcomes. Underpinning all these efforts, establishing robust standardization and quality control protocols for extraction, purification, and characterization is fundamental to ensuring product consistency, safety, and efficacy, thereby facilitating regulatory approval and successful clinical translation.

In conclusion, addressing these priorities through a concerted and interdisciplinary research effort will be crucial for overcoming the existing barriers. By systematically elucidating the mechanisms of action, defining clinical parameters, innovating delivery solutions, and embracing personalized medicine paradigms, the immense therapeutic potential of natural polysaccharides can be successfully translated into viable, effective, and safe clinical interventions for T2DM and its complications.

## Glossary

T2DM: Type 2 Diabetes Mellitus
DM: Diabetes mellitus
IDF: International Diabetes Federation
IR: Insulin Resistance
DAG: diacylglycerol
IL-17: Interleukin-17
IL-6: Interleukin-6
SOCS-3: Cytokine signaling inhibitors
IL-1β: Interleukin-1β
IL-18: Interleukin-18
NF-α: Tumor Necrosis Factor alpha
NF-κBp65: Nuclear Factor-κBp65
TSOD: T2DM mice
TSNO: Non-T2DM mice
SCFA: Short-Chain Fatty Acid
GPR43/41: G protein-coupled receptor43/41
GLP-1: Glucagon-like peptide-1
ROS: reactive oxygen species
NF-κB: oxidative stress induces nuclear factor κB
SAPK: stress-activated protein kinase
p38MAPK: p38 mitogen-activated protein kinase
PDX-1: Pancreatic and Duodenal Homeobox one
FOXO: Forkhead box protein O transcription factor
MafA: Transcription factor A
DAG: diacylglycerol
PINK1/Parkin: Homologous phospho-tensin-inducible kinase 1/parkinson protein
PI3K/AKt: Phosphatidylinositol 3-kinase/protein kinase B
GLUT4: Glucose transporter protein 4
PI3K: Phosphatidylinositol 3-kinase
IRS, IRS-1: Insulin receptor substrate; Insulin receptor substrate-1
AKt: protein kinase
ITP: Isatis tinctoria polysaccharide
PLPE: Phellinus linteus polysaccharide
SCAP: Schisandra chinensis acid polysaccharide
CRP: C Reactive proteins
P-JNK: Phosphorylated c-Jun terminal kinase
P-IRS-1: Phosphorylated insulin receptor substrate-1
MLP: Mulberry Leaf polysaccharide
Bcl2: B-cell leukemia/lymphoma 2
GLUT2: Glucose transporter protein 2
GCK: glucokinase
HOMA-IR: Homeostasis Model Assessment for insulin resistance
YPS: Yam polysaccharide
GLP: Ganoderma lucidum polysaccharide
IGF-1: Insulin-like growth factor 1
APS: Astragalus polysaccharide
LPS: lipopolysaccharide
STZ: streptozotocin
GPP: Gynostemma pentaphyllum polysaccharide
ALP: alkaline phosphatase
ALT: alanine transaminase
AST: aspartic transaminase
BUN: blood urea nitrogen
SOD: superoxide dismutase
CAT: Catalase
GSH-Px: Glutathione peroxidase
MDA: malondialdehyde
IL-4: Interleukin-4
IL-10: Interleukin-10
SBP: Stachys Baicalensis polysaccharides
MCP-1: Monocyte Chemoattractant Protein-1
ICAM-1: intercellular cell adhesion molecule-1
LBP: Lycium barbarum Polysaccharide
MDSC: myeloid-derived suppressor cell
SCP: Schisandra chinensis polysaccharide
SGP: Siraitia grosvenorii
DN: diabetic nephropathy
CCPP: Cymbopogon schoenanthus polysaccharide
gpcr: g protein-coupled receptor
PYY: Peptide tyrosine-tyrosine
CSP: Camellia sinensis (L.) Kuntze polysaccharide
TMSP: Trichosanthes kirilowii Maxim polysaccharide
PPS: pumpkin polysaccharide
TC: total cholesterol
LDL: low density lipoprotein
HDL: high density lipoprotein
GMP: soybean polysaccharides
PSP: Polygonatum sibiricum polysaccharide
WCP: *Wolfiporia cocos* (F.A. Wolf) Ryvarden & Gilb. polysaccharide
SIRT1: silent message regulator1
ERK: Extracellular signal-regulated kinases
MPC5: Mouse renal peduncle cells 5
PMP: Polygoni Multiflori Polysaccharides
RPE: retinal pigment epithelial cells
MLP: Mulberry polysaccharide
CCO: cytochrome C oxidase
SDH: succinate dehydrogenase
LC3: microtubule-associated protein 1 light chain 3
Drp1: dynamin-related protein 1
ASP: Angelica polysaccharide
CAV1: Caveolin-1
SDHA: Succinate dehydrogenase subunit A
LNT: Lentinan
DR: diabetic retinopathy
MCP: Momordic acharantia polysaccharide
AQP1: Aquaporin 1
Gli1: Glioma-associated oncogene homologs 1
CGP: *Crassostrea gigas* polysaccharide
ESRD: end-stage renal disease
FBG: Fasting Blood Glucose
BUN: Blood Urea Nitrogen
Scr: serum creatinine
CCP: Cordyceps cicadae polysaccharides
ZMP: *Zea mays* L. polysaccharidesPolysaccharides
SAA3: Serum amyloid A3
DCM: Diabetic cardiopathy
BMP10: Bone morphogenetic protein10
BMPR2: Bone morphogenetic protein type II receptor
PERK: Protein kinase R-like endoplasmic reticulum kinase
DF: Diabetic foot
DFU: Diabetic foot ulcers
DFI: Diabetic Foot Infection
AVP: Aloe vera polysaccharide
BFGF: Basic Fibroblast Growth Factor
PDGF-BB: Platelet-derived growth factor-BB
GSK3β: Glycogen synthesis kinase-3β
BSP: Bletilla striata polysaccharide
PHP: Pseudostellaria Heterophylla Polysaccharide
Fib: fibroblast
